# Stability study of common vasoactive drugs diluted in five types of solutions

**DOI:** 10.3389/fphar.2025.1670183

**Published:** 2025-09-19

**Authors:** Han Yang, Bingbing Xiang, Deiying Gong, Guoyan Zhao, Wensheng Zhang

**Affiliations:** ^1^ Department of Anesthesiology, West China Hospital, Sichuan University, Chengdu, China; ^2^ Laboratory of Anesthesia and Critical Care Medicine, National-Local Joint Engineering Research Centre of Translational Medicine of Anesthesiology, West China Hospital, Sichuan university, Chengdu, China

**Keywords:** vasoactive drugs, 0.9% sodium chloride injection, sodium lactate Ringer’s injection, glucose sodium chloride injection, glucose injection, stability, high-performance liquid chromatography

## Abstract

**Background:**

Vasoactive drugs are widely used during the perioperative period. Different vasoactive drugs have specific recommended solutions for dilution as stated in their instructions, but non-recommended solutions are sometimes used in clinical practice. The impact of using non-recommended solutions on drug stability remains unclear. This study investigated the stability of various commonly used vasoactive drugs diluted with five commonly used solutions—0.9% sodium chloride injection, sodium lactate Ringer’s injection, glucose sodium chloride injection, 5% glucose injection, and 10% glucose injection—under room temperature (24 °C ± 1 °C) without light protection.

**Methods:**

Each drug was diluted to clinically common concentrations using the five solutions mentioned above. Five samples of 100 µL each were prepared for each drug. The samples were stored at room temperature without light protection and observed at 0, 2, 4, 6, and 8 h for changes in appearance and pH. High-performance liquid chromatography (HPLC) was used to measure the drug content at each time point. The drug content at 0 h was set as 100%, and the content at other time points was calculated relative to this baseline.

**Results:**

Within 8 h, all solutions remained clear and transparent. Except for amiodarone hydrochloride, nicardipine hydrochloride, propafenone hydrochloride, and diltiazem hydrochloride, which showed significant pH changes after dilution, the pH changes of the other solutions were less than 0.1. Except for isoproterenol hydrochloride, the content of the other tested drugs showed no significant differences within 8 h.

**Conclusion:**

When diluted with the five commonly used solutions and stored at room temperature without light protection for 8 h, the tested drugs maintained stable properties.

## Introduction

The use of vasoactive drugs during the perioperative period is very common. Different vasoactive drugs have specific recommended solutions for dilution as per their instructions, such as adrenaline which is recommended to be prepared with 0.9% sodium chloride injection or 5% glucose injection, and amiodarone which can only be prepared with 5% glucose solution, and so on. However, in clinical practice, there are instances where non-recommended solutions are used for drug preparation. A study reveals that during the preparation of intravenous medications, the incidence of incorrect solution mixing stands at approximately 2% (Parshuram et al., 2008). The use of different liquids for drug reconstitution has a certain impact on drug stability and efficacy (Xu et al., 2025). However, the effect of preparing vasoactive drugs with non-recommended liquids on drug stability remains unclear at present. This experiment was conducted to investigate the stability of various drugs in different solutions, encompassing adrenaline hydrochloride, noradrenaline bitartrate, isoprenaline hydrochloride injection, dopamine hydrochloride injection, dobutamine hydrochloride injection, metaraminol bitartrate injection, milrinone injection, diltiazem hydrochloride for injection, nicardipine hydrochloride injection, urapidil hydrochloride injection, amiodarone hydrochloride injection, and propafenone hydrochloride injection. The drugs were diluted to clinically relevant concentrations using five commonly employed solutions: 0.9% sodium chloride injection, lactated Ringer’s injection, glucose and sodium chloride injection, 5% glucose injection, and 10% glucose injection. Subsequently, the stability of these drugs was evaluated under room temperature conditions (24 °C ± 1 °C) without light protection. This experiment was designed to simulate the scenario in operating rooms where vasoactive drugs are prepared for prompt use in rescuing patients, thereby providing an objective basis for guiding clinical medication.

## Methods and materials

### Chemicals and reagents

Trifluoroacetic acid (HPLC grade) was purchased from Sigma-Aldrich Co., Ltd. (MO, United States). Acetonitrile and methanol were of HPLC grade and purchased from Chengdu Nuoershi Technology Co., Ltd. (Chengdu, China). Ultrapure water was obtained using a Milli-Q integral water purification system (Merck Millipore, Darmstadt, Germany).^®^ Isoprenaline Hydrochloride, urapidil, milrinone and propafenone hydrochloride were acquired from the China National Institutes for Food and Drug Control. Adrenaline, L-Noradrenaline, dopamine, dobutamine hydrochloride, metaraminol bitartrate, diltiazem, amiodarone and nicardipine were purchased from CATO Research Chemicals Inc. (Guangzhou, China).

## Drugs

0.9% Sodium Chloride Injection, sodium Lactate Ringer’s Injection, Glucose and Sodium Chloride Injection, 5% Glucose Injection and 10% Glucose Injection were all purchased from Qingshan Likang Pharmaceutical Co., Ltd. (Chengdu, China). The vasoactive drugs used are shown in Table 1.

**TABLE 1:** Drug name and brand name (manufracturer).

Drug name	Brand name (manufacturer)
Adrenaline Hydrochloride	Xi’an Lijun Pharmaceutical Co., Ltd
Noradrenaline Bitartrate	Tianjin Jin Yao Pharmaceutical Co., Ltd
Isoprenaline Hydrochloride Injection	Southwest Pharmaceutical Co., Ltd
Dopamine Hydrochloride Injection	Anhui Changjiang Pharmaceutical Co., Ltd
Dobutamine Hydrochloride Injection	Guangdong Nanguo Pharmaceutical Co., Ltd
Metaraminol Bitartrate Injection	Nanjing Zeheng Pharmaceutical Technology Development Co., Ltd
Milrinone Injection	Hainan Herui Pharmaceutical Co., Ltd
Diltiazem Hydrochloride for Injection	Tianjin Tanabe Pharmaceutical Co., Ltd
Nicardipine Hydrochloride Injection	Astellas Pharma (China) Co., Ltd
Urapidil Hydrochloride Injection	Jiangxi Qingfeng Pharmaceutical Co., Ltd
Amiodarone Hydrochloride Injection	Shandong Beida Gaoke Huatai Pharmaceutical Co., Ltd
Propafenone Hydrochloride Injection	Guangzhou Baiyunshan Mingxing Pharmaceutical Co., Ltd

### Chromatographic conditions

Instrument: Shimadzu LC-20AD High-Performance Liquid Chromatograph from Shimadzu Co., Ltd. (Japan); Column: Swell Chromplus (4.6*250 mm, 5 μm) from Swell Scientific Instruments Co., Ltd (Chengdu, China), Flow rate: 1 mL/min, Isocratic elution. Needle wash: MeOH-H2O (1:1, v/v). The concentration of each drug was calculated based on peak area. The mobile phase, detection wavelength, and injection volume are shown in Table 2.

**TABLE 2:** Drug concentrations and chromatographic conditions.

Drug	Concentration (μg/mL)	Chromatographic conditions		
		Volume (μL)	Wavelength (nm)	Mobile phase
EPI	100	10	279	0.1%TFA:MeOH = 95:5
NE	100	10	279	0.1%TFA:MeOH = 95:5
ISO	50	10	279	0.1%TFA:ACN = 95:5
DA	1,000	2	280	0.1%TFA:MeOH = 85:15
DOB	200	10	279	0.1%TFA:ACN = 75:25
MET	1,000	1	273	0.1%TFA:ACN = 91:9
MIL	100	10	323	0.1%TFA:MeOH = 85:15
DIL	1,000	2	237	0.1%TFA:ACN = 55:45
NIC	100	10	237	0.1%TFA:ACN = 57:43
URA	1,000	5	264	0.1%TFA:ACN = 73:27
AMIO	1,000	2	240	0.1%TFA:ACN = 30:70
PRO	500	10	247	0.1%TFA:ACN = 53:47

Abbreviation: EPI: epinephrine hydrochloride; NE: norepinephrine bitartrate; ISO: isoproterenol hydrochloride; DA: dopamine hydrochloride; DOB: dobutamine hydrochloride; MET: metaraminol bitartrate; MIL: milrinone; DIL: diltiazem hydrochloride; NIC: nicardipine hydrochloride; URA: urapidil hydrochloride; AMIO: amiodarone hydrochloride; PRO: propafenone hydrochloride; TFA: trifluoroacetic acid; ACN: acetonitrile; MeOH: methanol.

### Specificity investigation

Reference Standard Solution: Accurately weigh an appropriate amount of each reference standard using a balance and prepare to the target concentration listed in Table 2 using the mobile phase as the reference stock solution.

Negative Control Solutions: 0.9% sodium chloride injection, sodium lactate Ringer’s injection, glucose sodium chloride injection, 5% glucose injection, and 10% glucose injection.

Test Solutions: Prepare each test drug to the target concentration listed in Table 2 using 0.9% sodium chloride injection, sodium lactate Ringer’s injection, glucose sodium chloride injection, 5% glucose injection, and 10% glucose injection, respectively.

**FIGURE 1:**
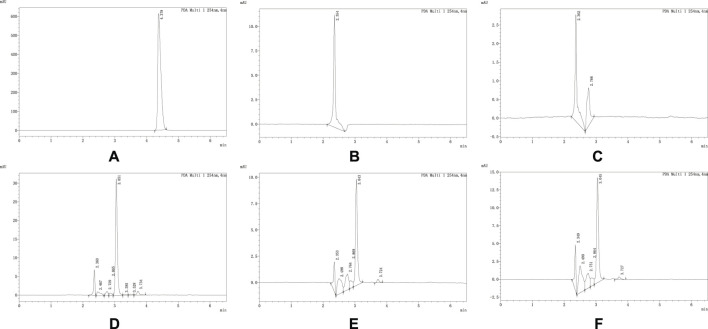
**(A–F)** respectively represent the chromatograms of propafenone hydrochloride reference standard, 0.9% Sodium Chloride Injection, Sodium Lactate Ringer’s Injection, Glucose Sodium Chloride Injection, 5% Glucose Injection and 10% Glucose Injection obtained under the chromatographic conditions corresponding to propafenone. As shown in the figure, the retention time of the propafenone hydrochloride reference standard is 4.378 min. No interfering peaks were observed at this time point in other samples, indicating that under these chromatographic conditions, other components in the solutions do not interfere with the determination of propafenone hydrochloride. The same applies to other drugs, and the chromatograms are shown in the attached figures.

Inject 100 μL of the reference standard solution, negative control solution, and test solution according to the chromatographic conditions specified in “Table 2” to observe the specificity of each drug peak, as shown in Figure 1. All solutions should be prepared fresh before use.

### Precision and repeatability investigation

Inject each drug reference standard solution in “Specificity investigation” section six times consecutively, and calculate the relative standard deviation (RSD) of the peak areas for each component as the precision. Meanwhile, prepare six test solutions in parallel using the drug to be tested and the mobile phase, with the preparation concentrations as specified in Table 2. The RSD of the peak areas for each component is then calculated as the repeatability (Matuszewski et al., 2003). The RSD for precision and repeatability are accepted within ±20% (U.S. Food and Drug Administration, 2018).

### Stability investigation of each drug in combination with five common solutions

Take the test samples prepared from the five solutions mentioned in “Specificity investigation” section and store them at room temperature without light protection. Observe the appearance changes of the solutions at 0, 2, 4, 6, and 8 h, respectively. Measure the pH values of the solutions using a pH meter, SevenDirect SD20, from Mettler-Toledo Instruments (Shanghai) Co., Ltd., and perform content determination according to the chromatographic conditions described in Table 2.

## Results

### Specificity

The experimental results showed that no interfering peaks were observed at the retention times of the drugs in the negative control solutions, indicating that under the conditions specified in Table 2, other substances in the test solutions did not interfere with the determination of the drugs.

### Precision and repeatability

The precision evaluation revealed that the relative standard deviations (RSDs) of the peak areas for epinephrine, norepinephrine, isoproterenol, dopamine, dobutamine, metaraminol, milrinone, diltiazem, nicardipine, urapidil, amiodarone, and propafenone were 0.16%, 0.13%, 0.17%, 1.38%, 0.09%, 0.30%, 0.08%, 0.52%, 0.73%, 1.20%, 0.16%, and 0.11%, respectively. In the repeatability assessment, the RSDs of the peak areas for the aforementioned 12 drugs were 0.27%, 0.48%, 0.17%, 0.37%, 0.19%, 0.63%, 1.18%, 0.43%, 0.59%, 0.47%, 1.02%, and 0.18%, respectively. The consistently low RSD values demonstrate that the method employed in this study exhibits excellent precision and repeatability.

### Drug stability investigation

All solutions were maintained clarity and transparency throughout the observation period, with no visible particulate matter detected by the naked eye. During this period, aside from amiodarone hydrochloride, nicardipine hydrochloride, propafenone hydrochloride, and diltiazem hydrochloride—which demonstrated significant pH variations following partial compatibility—changes in the pH of the remaining solutions were less than 0.1. The measured pH values and alterations in drug content are summarized in Table 3. With the exception of isoproterenol, the content of all other tested drugs showed no statistically significant differences within the 8-h timeframe.

**TABLE 3:** PH value and content change.

Drug		PH value					Content change (%)				
		0h	2h	4h	6h	8h	0h	2h	4h	6h	8h
EPI	NS	4.03	4.02	4.04	4.01	4.03	100.00	99.69	99.75	100.16	100.46
	LR	5.59	5.59	5.57	5.56	5.56	100.00	99.89	99.58	100.02	100.21
	GNS	3.91	3.91	3.94	3.94	3.93	100.00	100.20	99.70	100.31	101.44
	D5W	3.80	3.76	3.81	3.80	3.81	100.00	102.07	102.00	102.20	102.46
	D10W	3.60	3.59	3.66	3.67	3.69	100.00	103.58	103.94	104.36	105.70
EI	NS	3.84	3.84	3.84	3.85	3.85	100.00	100.40	100.15	99.68	99.21
	LR	5.60	5.60	5.60	5.63	5.61	100.00	100.22	99.99	94.98	98.77
	GNS	3.83	3.82	3.83	3.83	3.83	100.00	100.11	100.15	97.86	100.31
	D5W	3.81	3.81	3.81	3.82	3.80	100.00	99.92	99.95	98.01	100.52
	D10W	3.65	3.65	3.66	3.67	3.65	100.00	100.32	100.04	95.26	100.58
ISO	NS	4.18	4.14	4.16	4.17	4.17	100.00	100.62	100.34	\	100.43
	LR	5.61	5.58	5.58	5.56	5.56	100.00	100.28	100.09	\	100.16
	GNS	3.91	3.93	3.94	3.95	3.94	100.00	101.38	101.35	\	101.34
	D5W	3.85	3.82	3.86	3.87	3.87	100.00	88.44	88.53	\	88.66
	D10W	3.65	3.59	3.66	3.67	3.68	100.00	82.75	82.78	\	82.94
DA	NS	4.56	4.55	4.53	4.64	4.62	100.00	100.27	100.52	100.80	100.97
	LR	5.95	5.93	5.94	5.96	5.93	100.00	100.11	99.85	100.27	100.37
	GNS	4.11	4.13	4.15	4.15	4.16	100.00	100.23	99.99	100.22	100.21
	D5W	3.90	3.91	3.92	3.93	3.92	100.00	99.73	99.75	100.11	100.25
	D10W	3.67	3.68	3.68	3.69	3.71	100.00	100.06	99.85	100.09	100.02
DOB	NS	4.52	4.50	4.54	4.51	4.53	100.00	100.40	100.49	100.83	100.84
	LR	6.03	6.00	6.02	6.03	6.01	100.00	100.41	100.24	100.76	100.70
	GNS	4.07	4.05	4.07	4.06	4.06	100.00	100.11	100.29	100.29	100.32
	D5W	3.93	3.92	3.92	3.95	3.93	100.00	100.10	100.56	100.47	100.46
	D10W	3.59	3.59	3.60	3.63	3.60	100.00	100.24	100.25	100.30	100.42
MET	NS	3.45	3.44	3.49	3.47	3.46	100.00	100.33	100.66	100.04	101.47
	LR	4.37	4.37	4.37	4.38	4.37	100.00	100.33	99.92	100.25	99.78
	GNS	3.43	3.42	3.44	3.44	3.44	100.00	100.35	99.67	100.12	100.25
	D5W	3.57	3.57	3.59	3.58	3.58	100.00	100.28	99.95	99.83	100.75
	D10W	3.54	3.45	3.56	3.56	3.55	100.00	101.44	101.03	100.65	100.99
MIL	NS	3.70	3.70	3.71	3.72	3.71	100.00	100.04	100.56	100.07	100.33
	LR	5.05	5.05	5.04	5.07	5.05	100.00	105.41	105.57	105.46	105.98
	GNS	3.65	3.65	3.64	3.66	3.67	100.00	100.21	100.10	100.12	100.40
	D5W	3.69	3.69	3.69	3.70	3.70	100.00	99.57	99.57	99.52	99.83
	D10W	3.62	3.61	3.62	3.62	3.62	100.00	99.95	100.02	100.11	100.23
DIL	NS	6.17	6.11	5.96	6.06	5.93	100.00	101.71	101.14	102.11	101.45
	LR	6.39	6.34	6.30	6.17	6.32	100.00	100.88	100.90	101.88	101.10
	GNS	4.39	4.48	4.40	4.41	4.40	100.00	100.10	100.28	99.64	99.78
	D5W	4.15	4.19	4.21	4.19	3.79	100.00	100.00	101.28	100.24	99.84
	D10W	3.36	3.78	3.79	3.78	3.42	100.00	100.54	100.72	100.15	99.72
NIC	NS	4.63	4.60	4.60	4.58	4.57	100.00	86.52	94.54	104.01	92.78
	LR	6.13	6.08	6.06	6.08	6.09	100.00	109.00	114.14	104.68	109.09
	GNS	4.04	4.05	4.05	4.05	4.03	100.00	100.41	106.26	102.23	95.69
	D5W	3.88	3.89	3.90	3.90	3.74	100.00	105.66	75.67	99.83	94.79
	D10W	3.60	3.59	3.59	3.59	3.53	100.00	100.11	99.34	96.66	76.07
URA	NS	5.96	5.95	5.95	5.96	5.92	100.00	100.05	100.02	99.97	99.67
	LR	6.05	6.04	6.03	6.02	6.02	100.00	99.87	100.26	100.08	100.11
	GNS	5.85	5.85	5.86	5.82	5.81	100.00	99.92	100.04	100.02	99.96
	D5W	6.09	6.05	6.08	6.06	6.05	100.00	100.03	100.14	100.10	99.96
	D10W	5.96	5.95	5.91	5.91	5.90	100.00	100.02	99.88	99.83	99.82
AMIO	NS	4.53	4.51	4.54	4.45	4.48	100.00	100.22	99.98	99.96	100.44
	LR	5.71	5.62	5.59	5.59	5.54	100.00	99.86	99.18	97.37	97.85
	GNS	4.03	4.03	4.04	4.04	4.02	100.00	98.60	99.47	98.59	99.89
	D5W	3.71	3.71	3.70	3.72	3.74	100.00	99.34	99.31	99.91	100.09
	D10W	3.51	3.51	3.50	3.51	3.49	100.00	99.91	100.14	99.95	100.55
PRO	NS	6.30	6.29	6.33	6.62	5.65	100.00	100.73	100.50	100.57	100.63
	LR	6.40	6.40	6.32	6.52	6.43	100.00	100.50	100.75	100.63	100.87
	GNS	4.47	4.34	4.37	4.40	4.51	100.00	100.46	100.42	100.57	100.54
	D5W	4.18	4.20	4.20	4.22	4.23	100.00	100.61	100.45	100.29	100.45
	D10W	3.78	3.79	3.83	3.81	3.81	100.00	100.31	100.38	100.51	100.24

Abbreviation: NS: 0.9% Sodium Chloride Injection; LR: Sodium Lactate Ringer’s Injection; GNS: glucose sodium chloride injection; D5W: 5% Glucose Injection; D10W: 10% Glucose Injection; EPI: epinephrine hydrochloride; NE: norepinephrine bitartrate; ISO: isoproterenol hydrochloride; DA: dopamine hydrochloride; DOB: dobutamine hydrochloride; MET: metaraminol bitartrate; MIL: milrinone; DIL: diltiazem hydrochloride; NIC: nicardipine hydrochloride; URA: urapidil hydrochloride; AMIO: amiodarone hydrochloride; PRO: Propafenone Hydrochloride. The drug content at 0 h is set as the baseline (100%). The content at other time points (2, 4, 6, and 8 h) is calculated as a percentage relative to the 0-h content.

## Discussion

The purpose of this experiment is to simulate the scenario in operating rooms where vasoactive drugs are prepared for prompt use in rescuing patients, and investigate the stability of various drugs when diluted with five commonly used solutions, providing a basis for clinical medication. Therefore, the experiments were conducted under room temperature and without protection from light, and the drug concentrations were set at clinically common levels to simulate real clinical conditions.

This study found that all the drugs involved in the experiment remained stable in different solutions, with no significant changes in their content. This result is consistent with previous research findings (Tremblay et al., 2008; Rawas-Qalaji et al., 2009; Van Matre et al., 2023; Peddicord et al., 1997; Kaushal et al., 2013; Baaske et al., 1996; Dupuis et al., 1997; Hoellein and Holzgrabe, 2012). As shown in Table 3, the content of isoprenaline hydrochloride changed by more than 10% after 2 hours when prepared with 5% glucose solution or 10% glucose solution. To further investigate this observation, we prepared a control solution of the same concentration and quantified the content changes based on the peak area of the control solution. The results, presented in Table 4, demonstrate that isoprenaline hydrochloride remains stable in glucose solutions. The observed discrepancies were attributed to incomplete mixing of the solution, a common issue encountered in clinical practice (Teunissen et al., 2020; Campino et al., 2016). Although we lost the data for isoprenaline hydrochloride after 6 hours of storage following dilution, the available 8-h data does not hinder our conclusion that its properties remain stable. 1n addition, amiodarone hydrochloride has a pH range of 2.5–4.0. Its structure contains a benzofuran group, which is prone to degradation in solution as the pH changes (Coelho et al., 2020). The pH of 5% glucose injection ranges from 3.2 to 6.5, while that of 0.9% sodium chloride injection is 4.5–7.0. Therefore, it is clinically recommended that amiodarone be compatible with 5% glucose injection for administration. Then, while the content of amiodarone hydrochloride prepared with lactated Ringer’s solution decreased by approximately 3% after 6 hours compared to the initial measurement (0 h), this change remains within the acceptable range as the drug concentration still maintained above 90% of the initial measurement (0 h) (Pietsch et al., 2022). Therefore, we still consider it to be stable in our experimental period.

**TABLE 4:** Changes in amiodarone content.

Solution	Content change (%)				
	0h	2h	4h	6h	8h
D5W	112.72	99.69	99.79	\	99.94
D10W	121.10	100.22	100.25	\	100.44

The content of the 50 μg/mL amiodarone standard is set as 100%; D5W: 5% Glucose Injection; D10W: 10% Glucose Injection.

When diluted with different solutions, the content of each drug exhibited minimal fluctuations over a certain period. This indicates that, for clinical applications, the changes in drug concentration remain small within a specific timeframe, ensuring that the intended therapeutic effect can be achieved and reducing the risk of efficacy variability. Additionally, the choice of dilution solution has a negligible impact on drug content, allowing clinicians to select the most appropriate solution based on the patient’s specific condition. Furthermore, even when the recommended solution specified in the drug instructions is not strictly followed, the efficacy of the drug is not compromised due to the use of an alternative solution.

The pH of a solution can change due to various factors, such as alterations in solute composition, temperature, humidity, and bacterial contamination. In this experiment, while the content of each drug remained stable, significant pH changes were observed in some solutions. For example, when propafenone hydrochloride injection was prepared with 0.9% sodium chloride solution, the pH change exceeded 0.5 after 8 h of storage, despite no noticeable change in the drug content. This suggests the possibility of bacterial contamination as a contributing factor and we should not use it anymore (Sandberg and Talbot, 2016). To put it another way, the prepared drugs may be subject to bacterial contamination when stored for an extended period. This necessitates a prior assessment of relevant risks before their use, so as to prevent associated adverse reactions such as infections. Meanwhile, the stability of drug storage can also be influenced by the material of the container used. Different container materials may have varying effects on the preservation of drug properties over time (Dupuis et al., 1997). Moreover, the pH of a solution is associated with venous endothelial injury. According to relevant studies, drugs with a pH > 9 or pH < 4 are categorized as high-risk ones, which have extremely strong irritation to veins (Manrique-Rodríguez et al., 2021). And some researchers think that pH value should be greater than 5, which is more protective for the patient (Pittiruti et al., 2023; Pinelli et al., 2025). Solutions with extreme pH can damage the integrity of venous endothelium, trigger inflammation or vascular wall rupture, and thus peripheral venous infusion of such drugs should be avoided to prevent severe complications (Borgonovo et al., 2023). However, in the context of emergency rescue for patients, an established central venous access is not always available. Thus, we should make every effort to avoid the use of solutions with extreme pH values in emergency situations. For example, when various drugs are prepared using D10W, as shown in Table 3, only the pH of URA exceeds 4, while the pH of all other drugs is less than 4. Alternatively, for high-risk patients before surgery, priority should be given to establishing central venous access or using peripheral venous catheters to prevent venous injury (Borgonovo et al., 2023; Nickel et al., 2024).

Additionally, during the storage of the drugs, we observed that propafenone hydrochloride injection formed crystals when stored at 4 °C, indicating that it is unsuitable for low-temperature storage. However, this study did not further investigate the changes in drug content after re-dissolution of the crystals. Relevant experiments will be conducted in the future to explore the effects of different temperature conditions on the properties and stability of the drug.

The experimental results demonstrate that when different solutions are used to dilute and administer vasoactive drugs in clinical practice, the composition of the solution does not cause significant changes in drug concentration. Therefore, the stability and efficacy of the drugs remain predictable within a certain timeframe. The findings of this study provide critical guidance for the selection of dilution solutions and dose adjustments. Clinicians can tailor the choice of solution based on individual patient differences and specific clinical conditions. However, this experiment only simulated the scenario in the operating room. Therefore, the experimental results have limited guiding significance for medication in the ward, and the range of drugs covered is relatively narrow. Subsequent relevant experiments will be carried out to enhance the universality of the experimental conclusions and further guide clinical medication.

## Conclusion

Adrenaline, noradrenaline, isoprenaline, dopamine, dobutamine, metaraminol, milrinone, diltiazem, nicardipine, urapidil, amiodarone, and propafenone maintained their stability for 8 h when diluted with 0.9% sodium chloride injection, lactated Ringer’s injection, glucose and sodium chloride injection, 5% glucose injection, and 10% glucose injection, respectively, and stored at room temperature without protection from light.

## Data Availability

The original contributions presented in the study are included in the article/Supplementary Material, further inquiries can be directed to the corresponding author.
