# Supplementary material for: Stability study of common vasoactive drugs diluted in five types of solutions
Source: Front Pharmacol. 2025 Sep 19;16:1670183. doi: 10.3389/fphar.2025.1670183 (PMC12492491; doi:10.3389/fphar.2025.1670183)
Supplement: Supplementary file 1 [file Supplementaryfile1.doc]

| 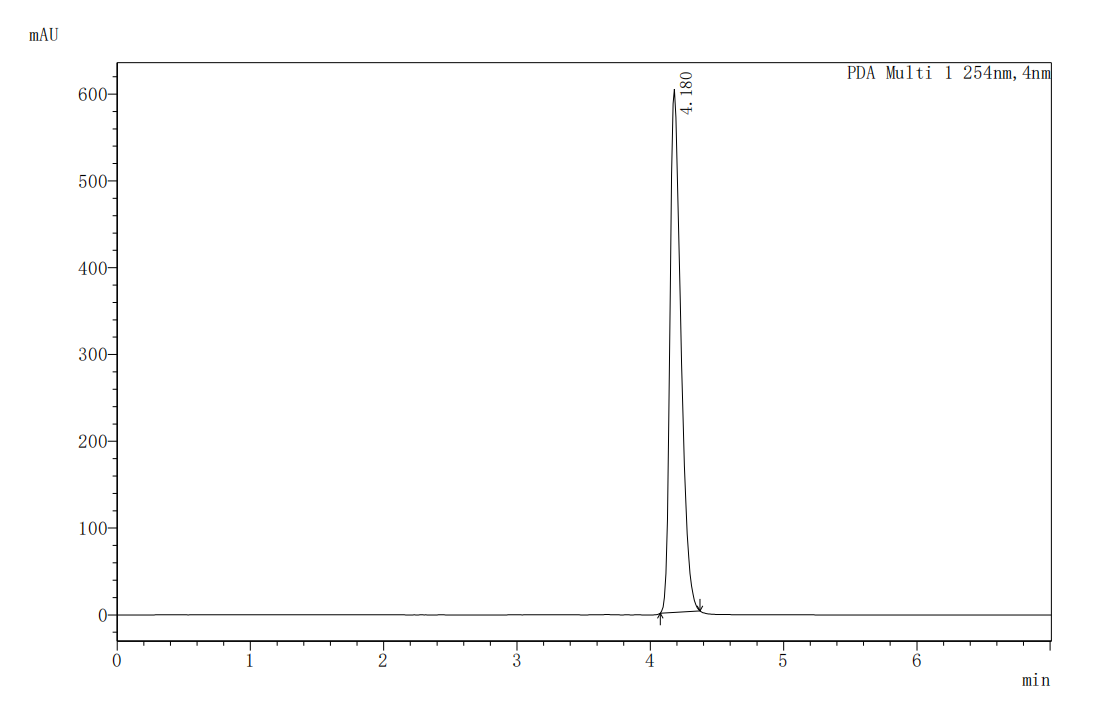A | 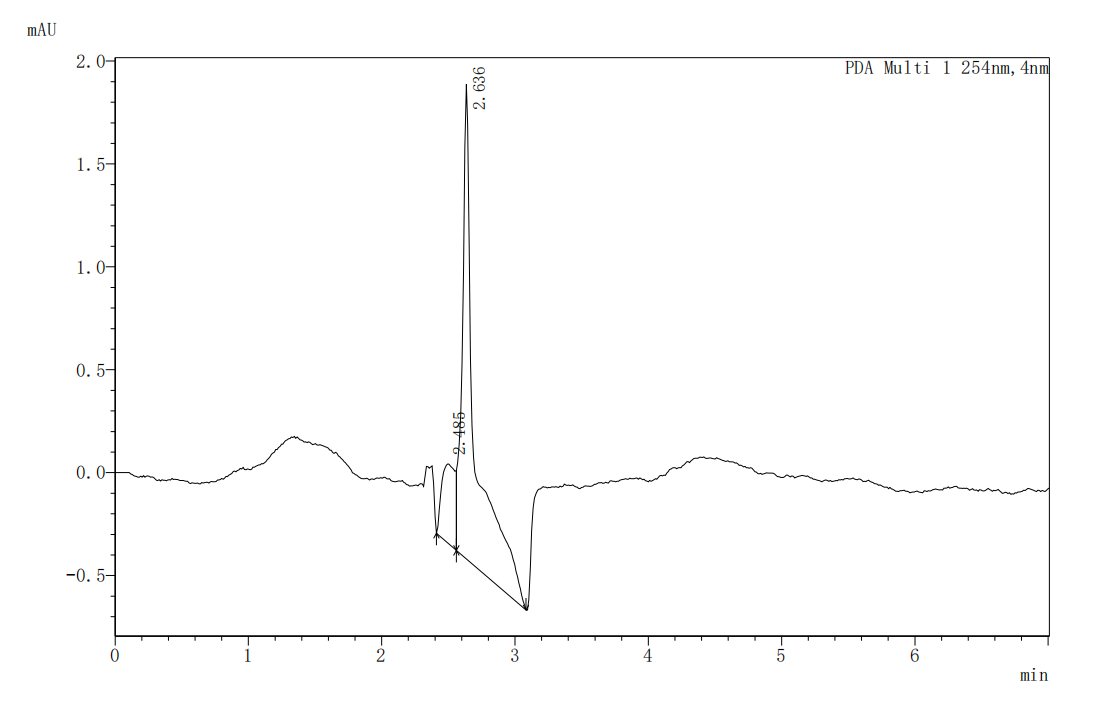B | 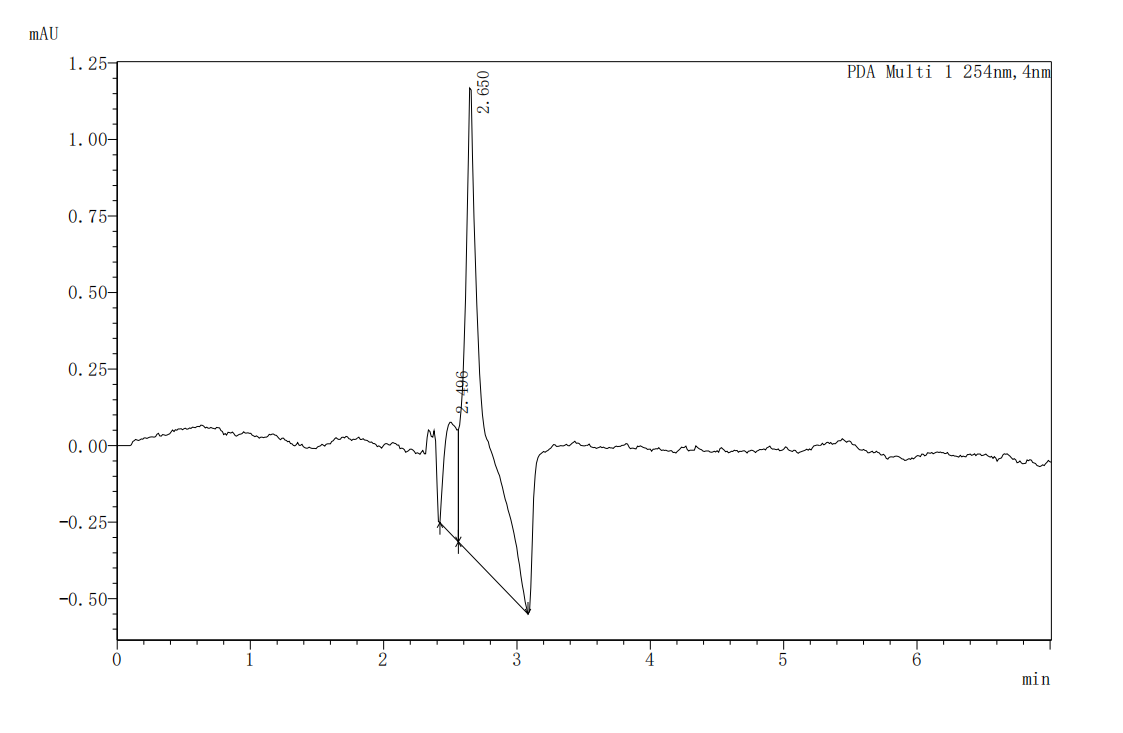C |
| --- | --- | --- |
| 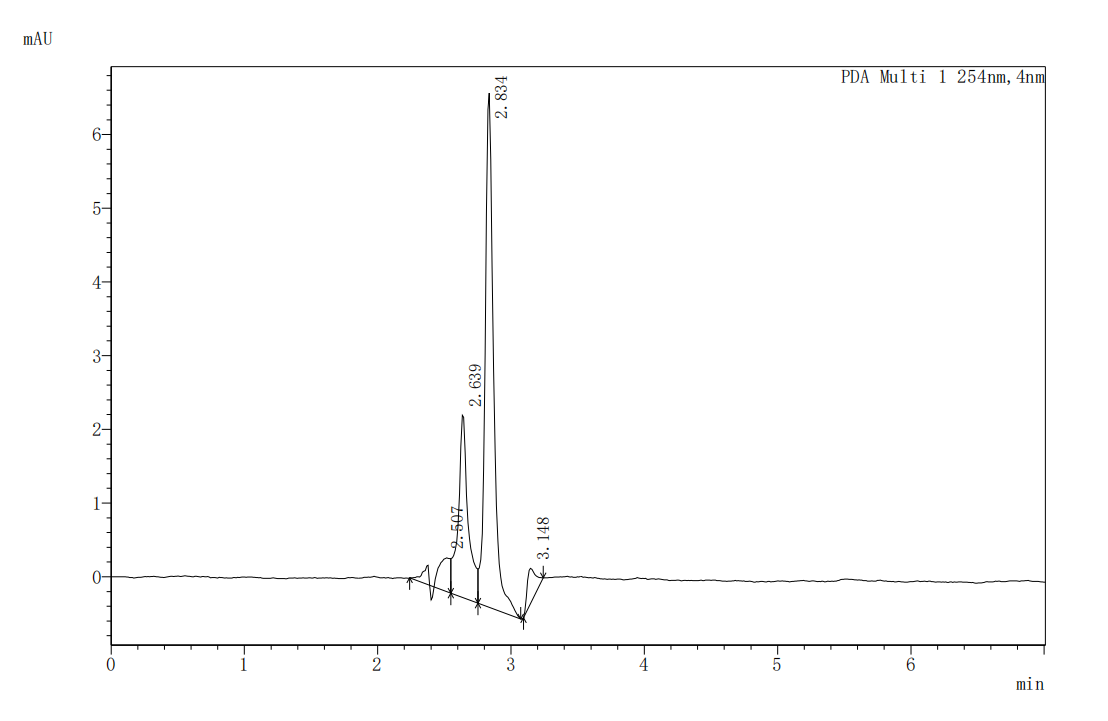D | 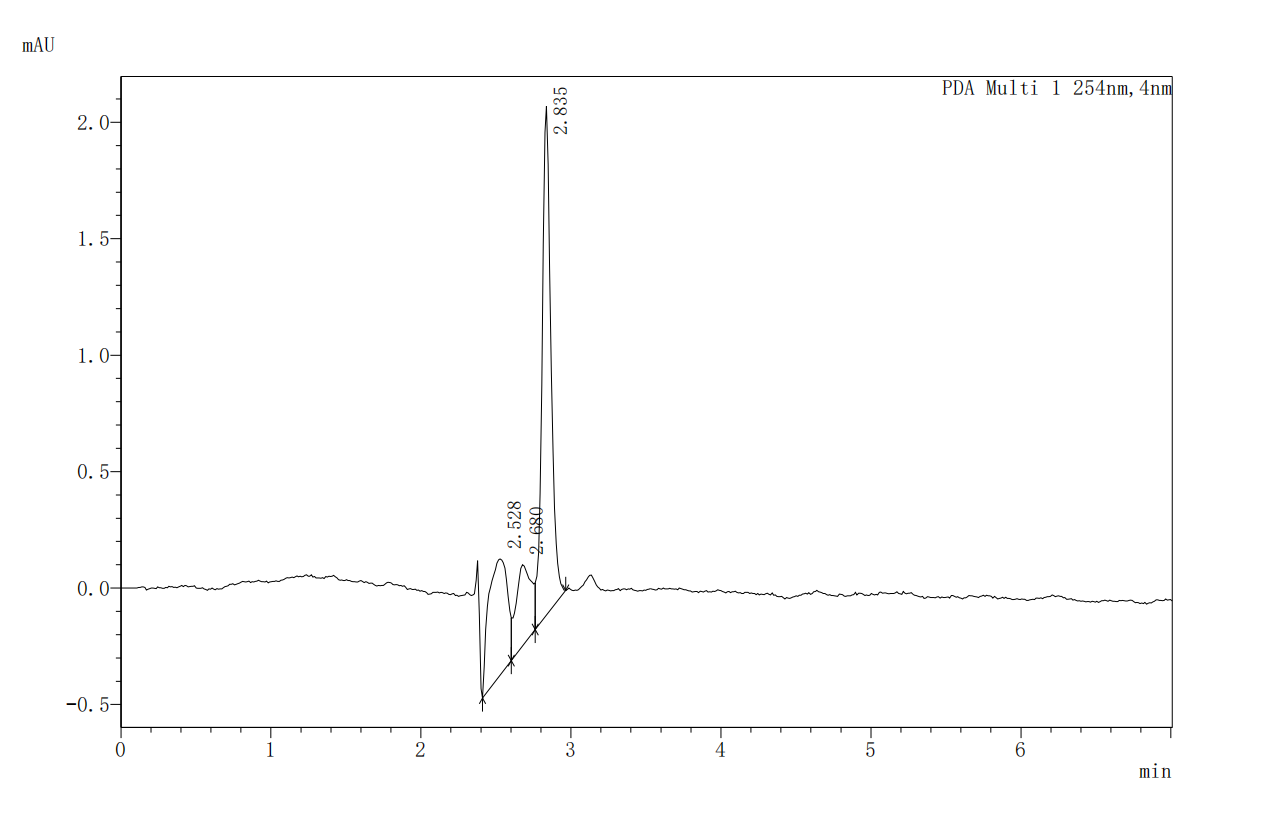E | 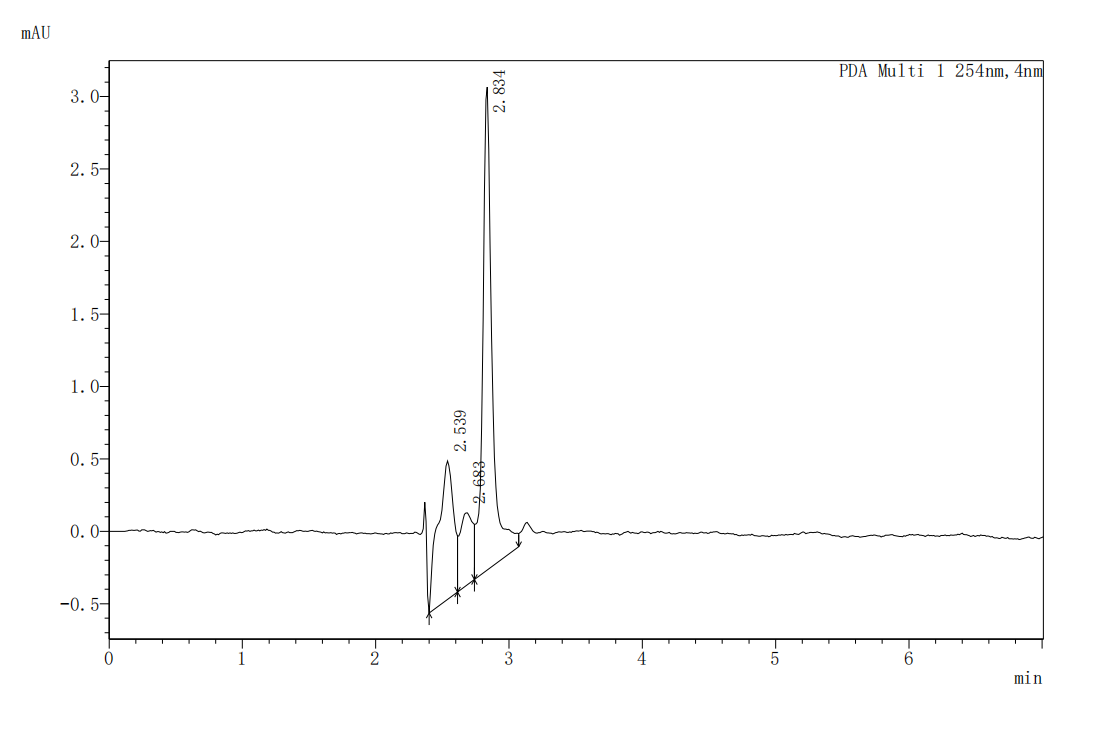F |

Figure S1 A, B, C, D, E, and F respectively represent the chromatograms of amiodarone hydrochloride reference standard, 0.9% Sodium Chloride Injection, Sodium Lactate Ringer's Injection, Glucose Sodium Chloride Injection, 5% Glucose Injection and 10% Glucose Injection obtained under the chromatographic conditions corresponding to amiodarone hydrochloride.

| 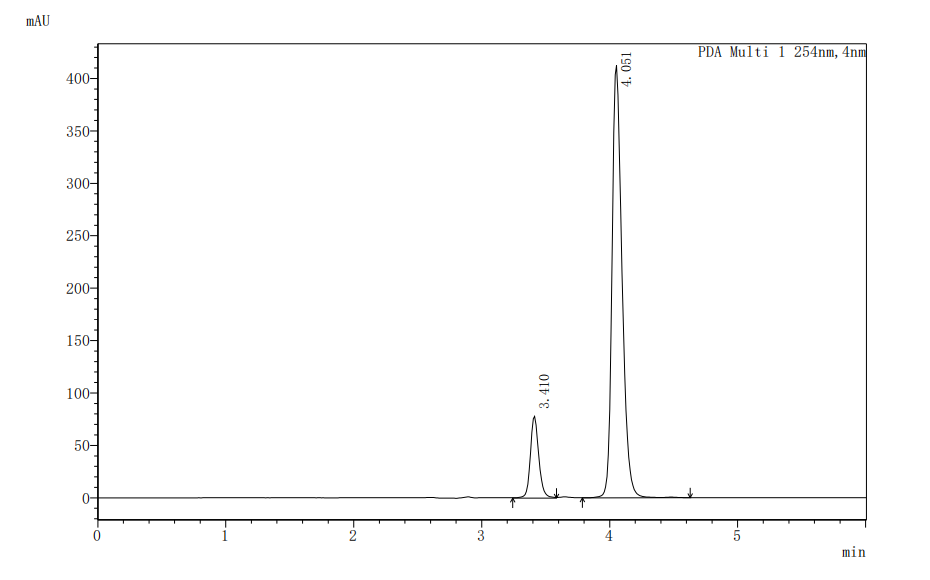A | 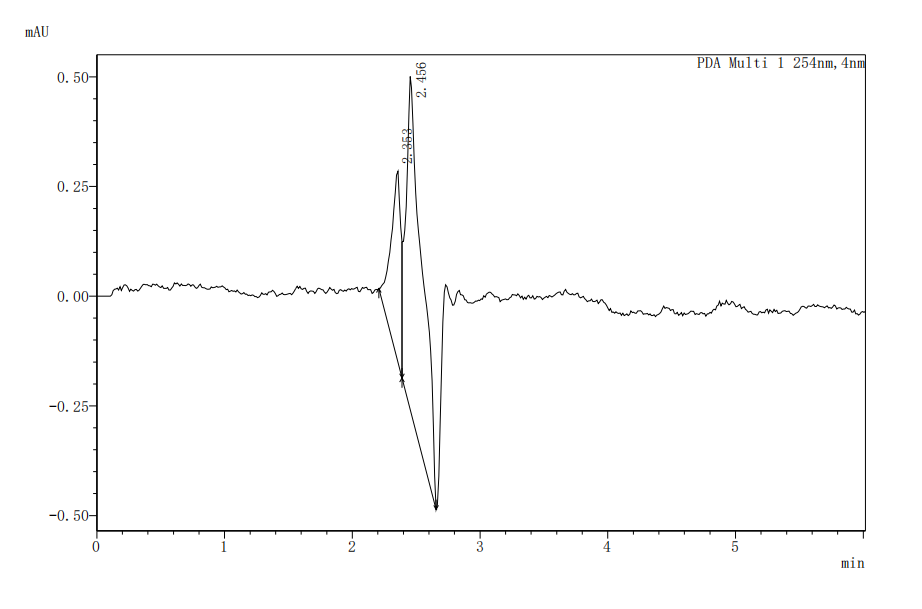B | 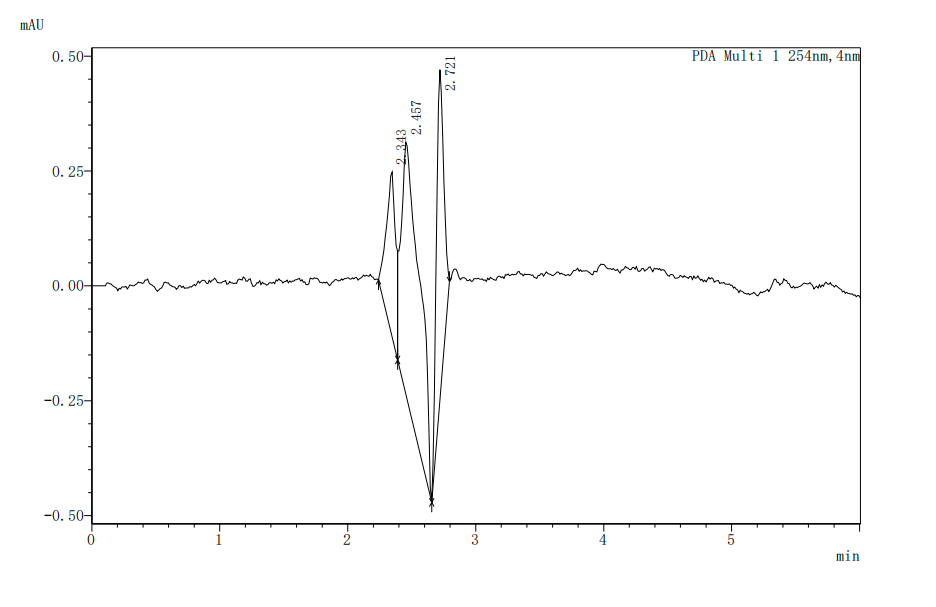C |
| --- | --- | --- |
| 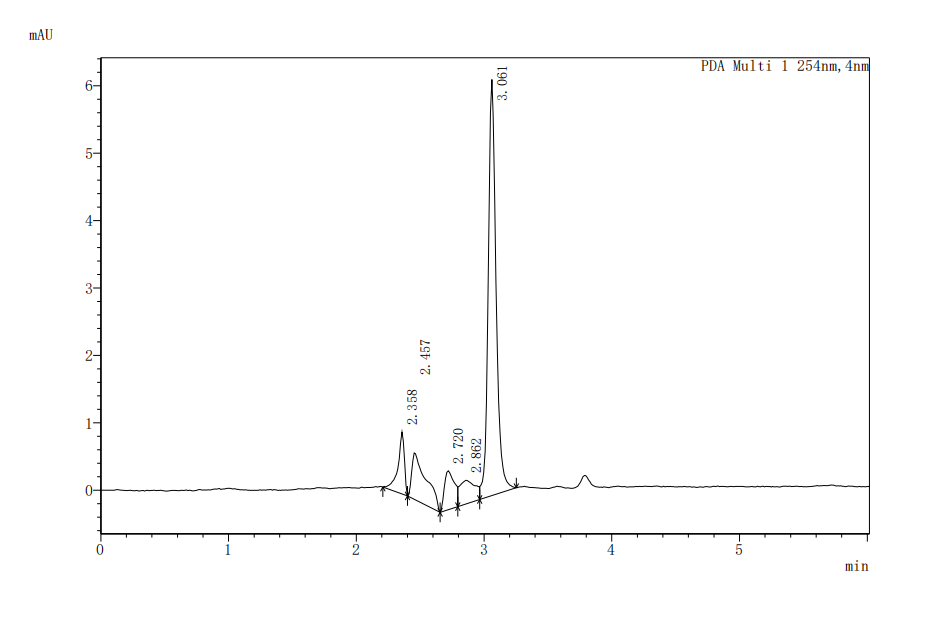D | 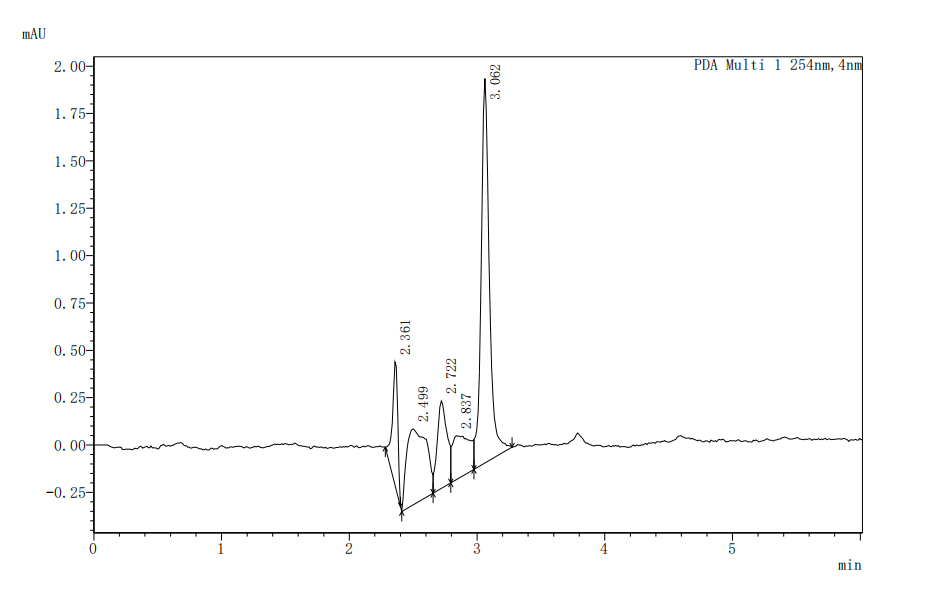E | 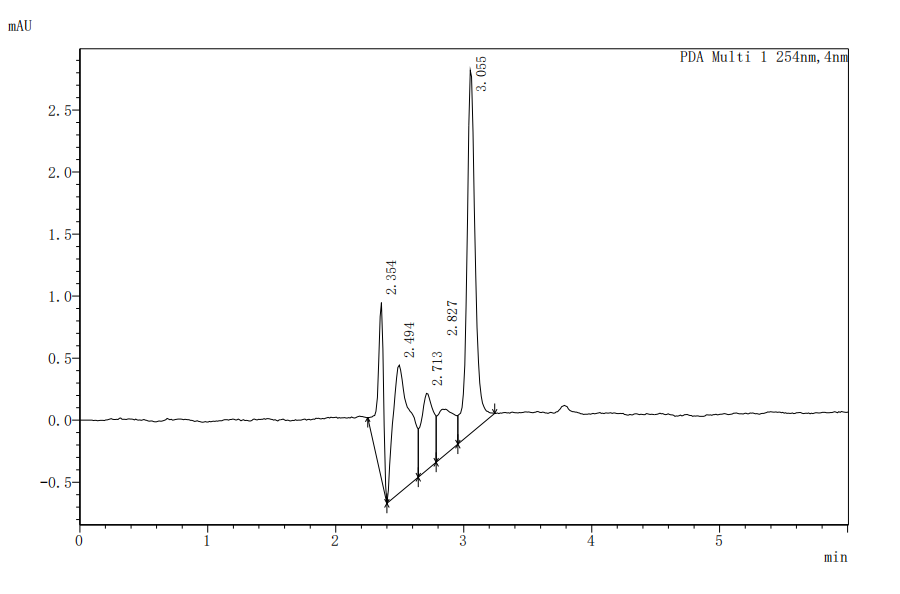F |

Figure S2 A, B, C, D, E, and F respectively represent the chromatograms of diltiazem hydrochloride reference standard, 0.9% Sodium Chloride Injection, Sodium Lactate Ringer's Injection, Glucose Sodium Chloride Injection, 5% Glucose Injection and 10% Glucose Injection obtained under the chromatographic conditions corresponding to diltiazem hydrochloride.

| 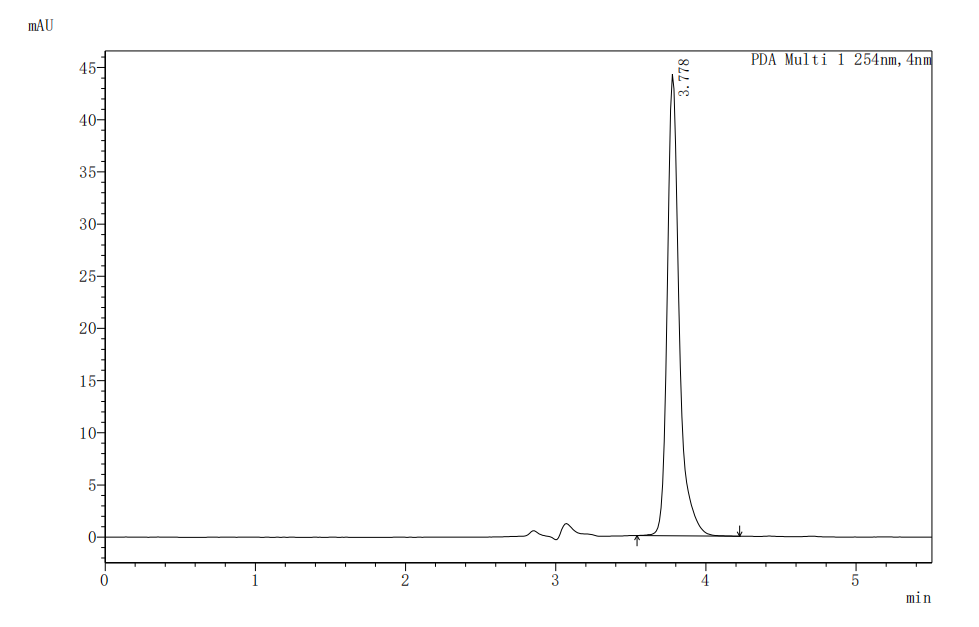A | 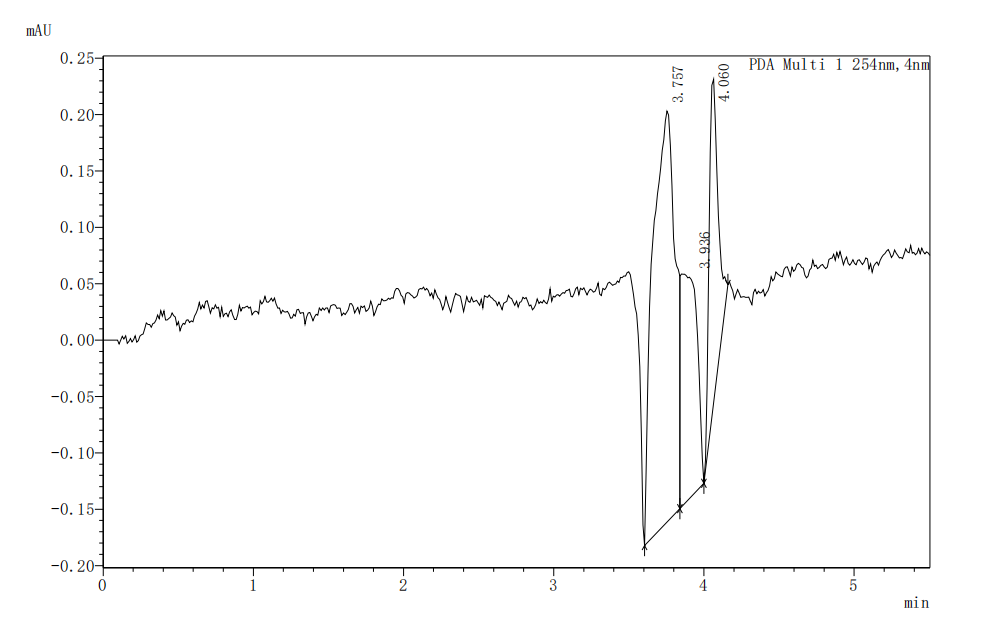B | 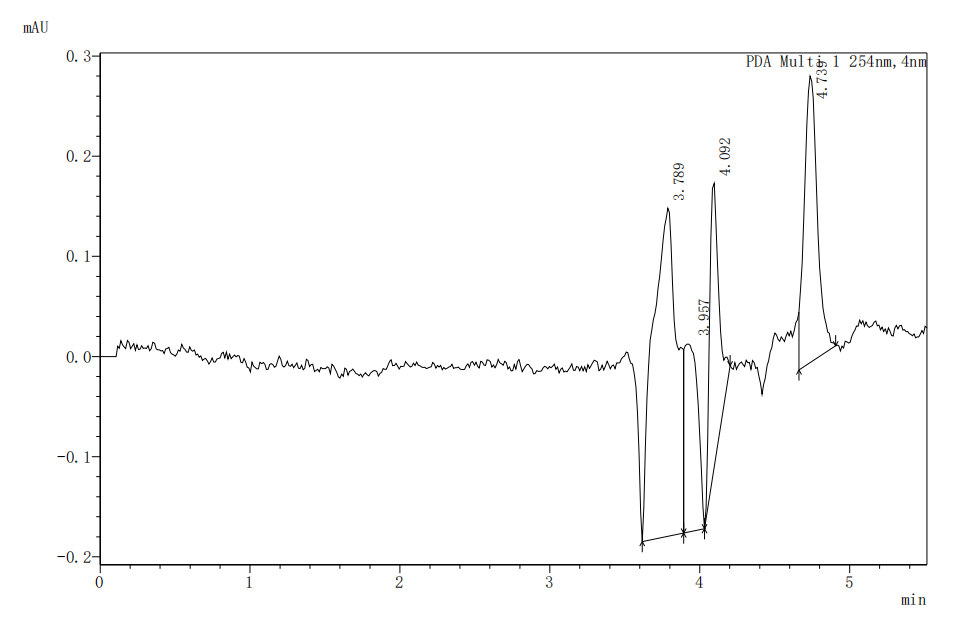C |
| --- | --- | --- |
| 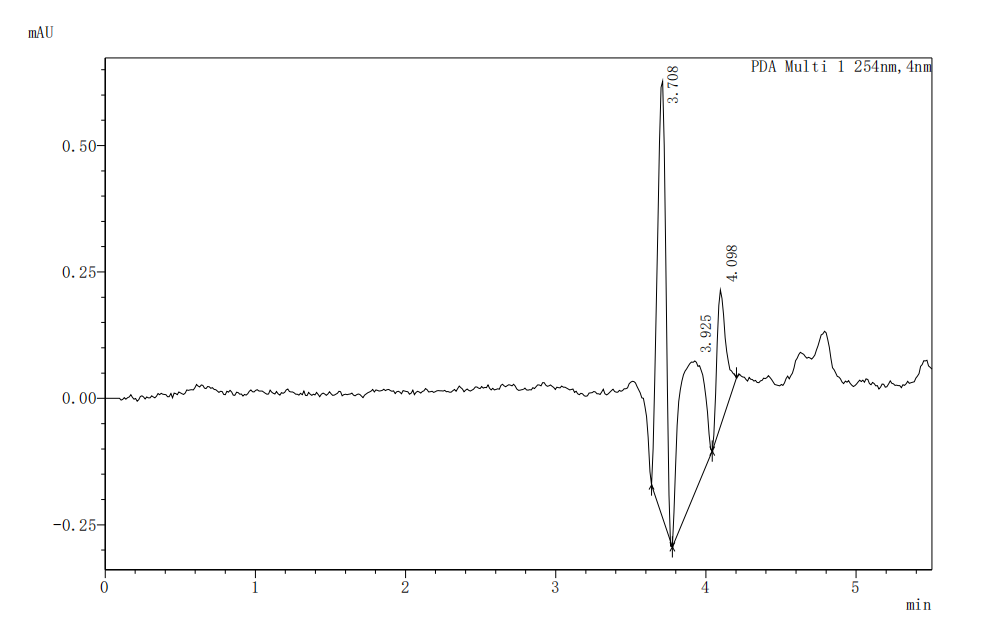D | 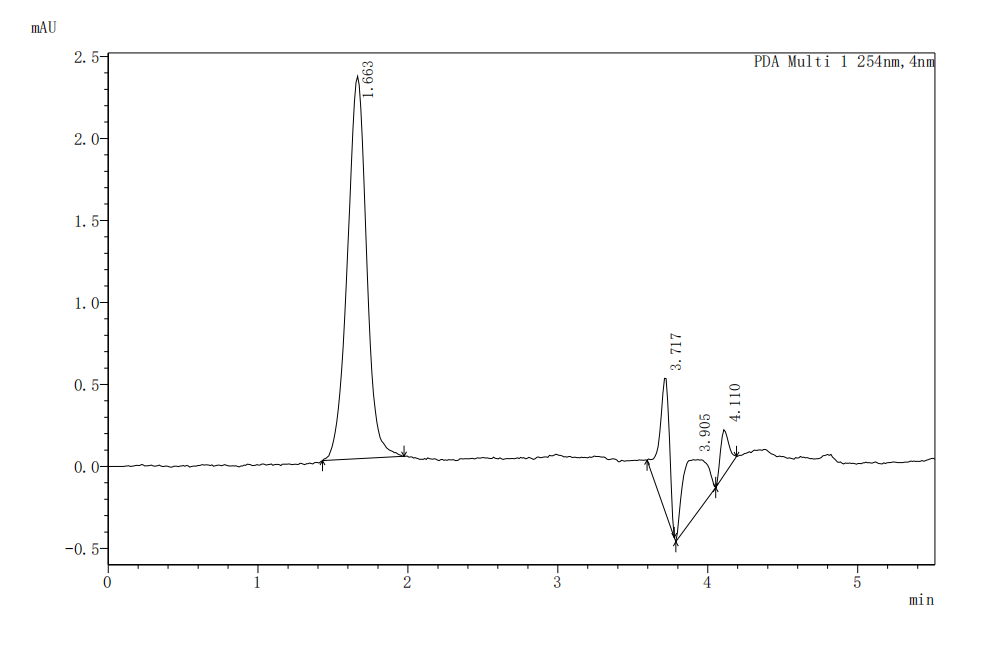E | 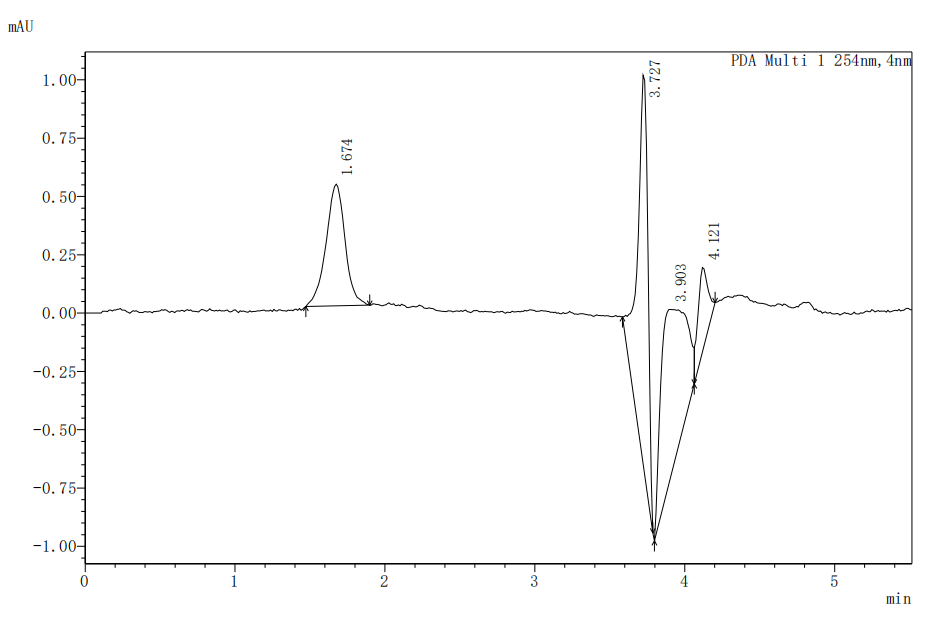F |

Figure S3 A, B, C, D, E, and F respectively represent the chromatograms of dopamine hydrochloride reference standard, 0.9% Sodium Chloride Injection, Sodium Lactate Ringer's Injection, Glucose Sodium Chloride Injection, 5% Glucose Injection and 10% Glucose Injection obtained under the chromatographic conditions corresponding to dopamine hydrochloride.

| 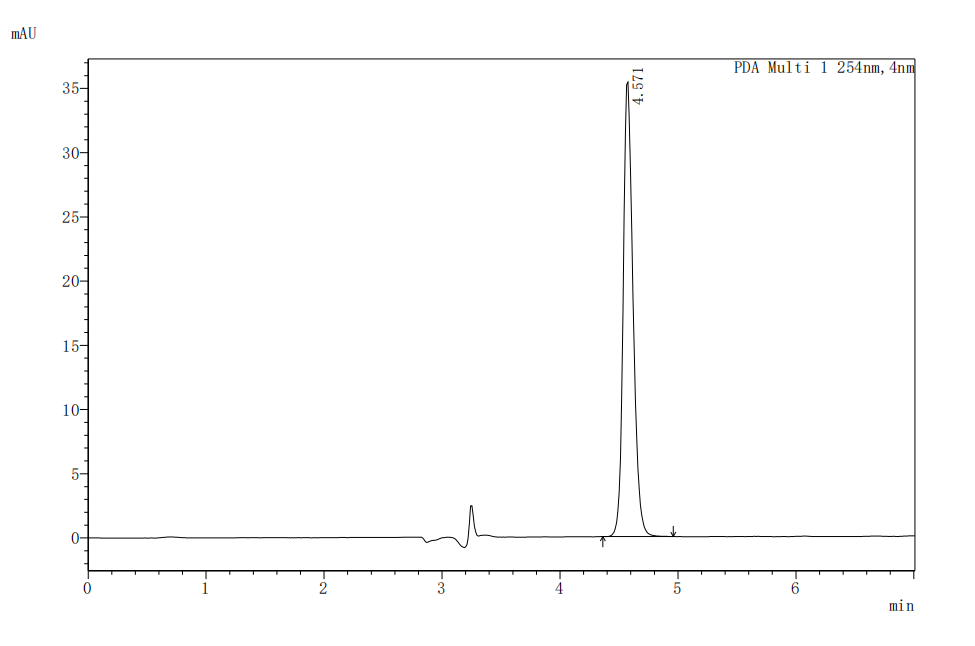A | 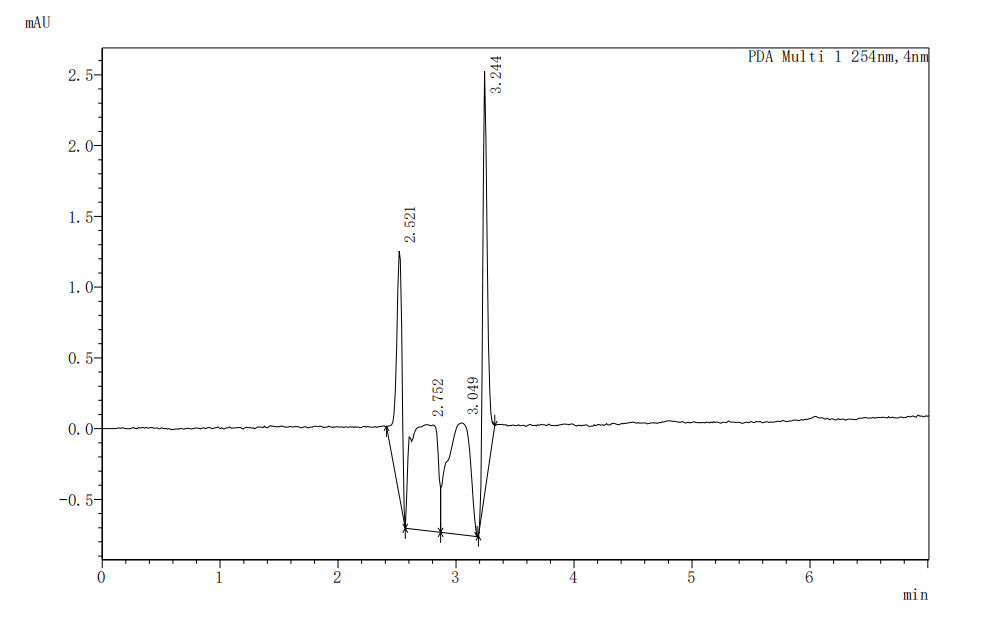B | 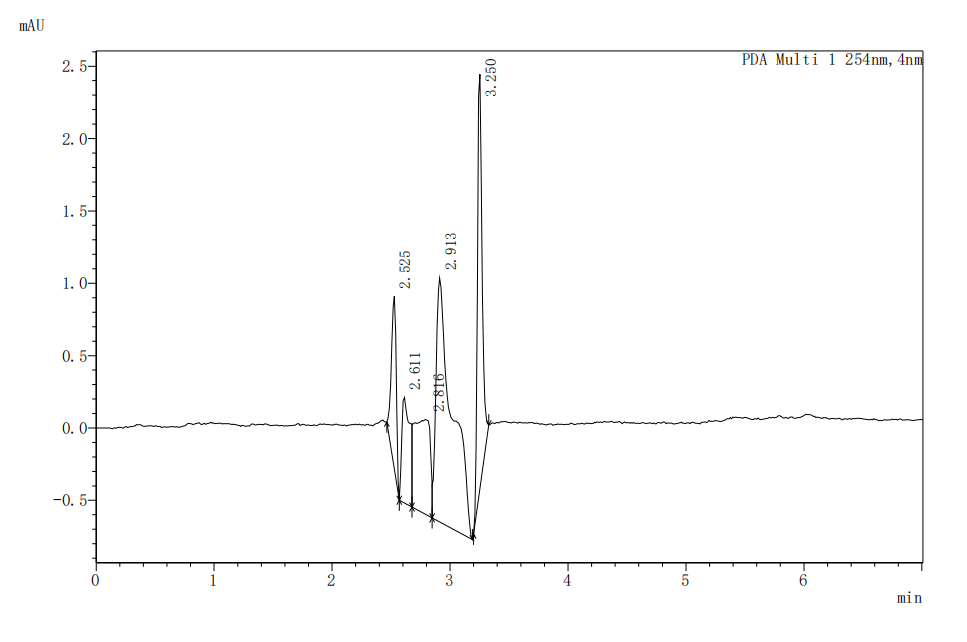C |
| --- | --- | --- |
| 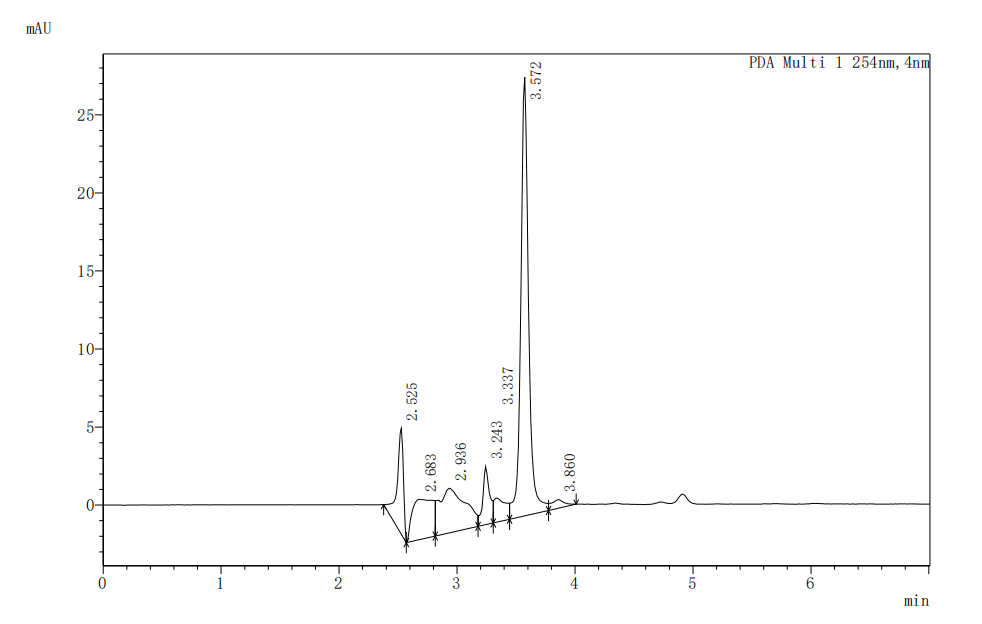D | 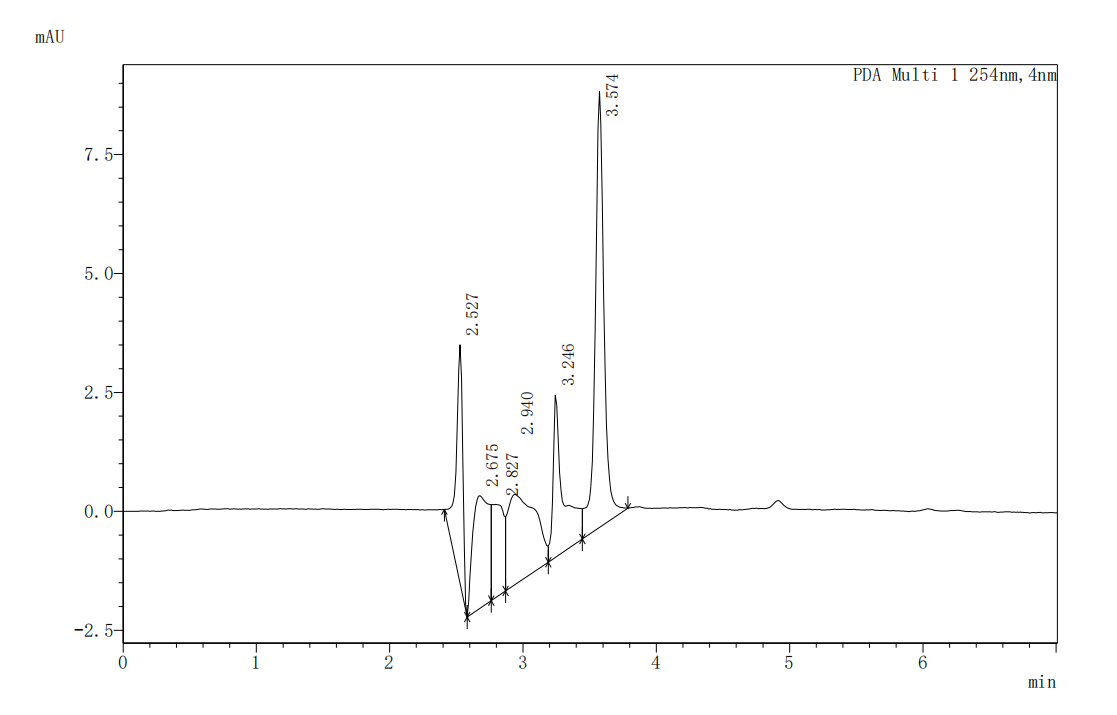E | 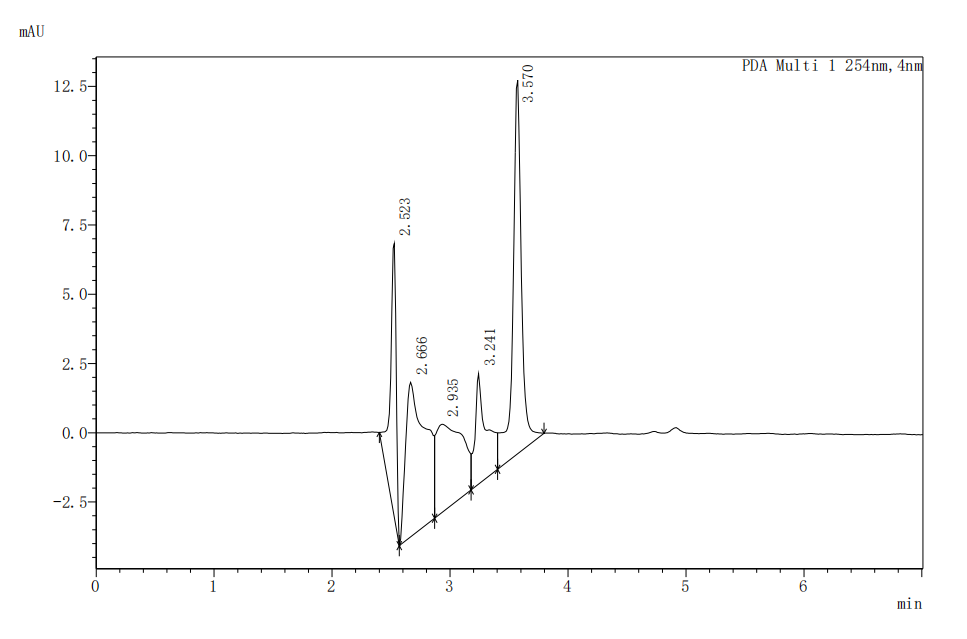F |

Figure S4 A, B, C, D, E, and F respectively represent the chromatograms of dobutamine hydrochloride reference standard, 0.9% Sodium Chloride Injection, Sodium Lactate Ringer's Injection, Glucose Sodium Chloride Injection, 5% Glucose Injection and 10% Glucose Injection obtained under the chromatographic conditions corresponding to dobutamine hydrochloride.

| 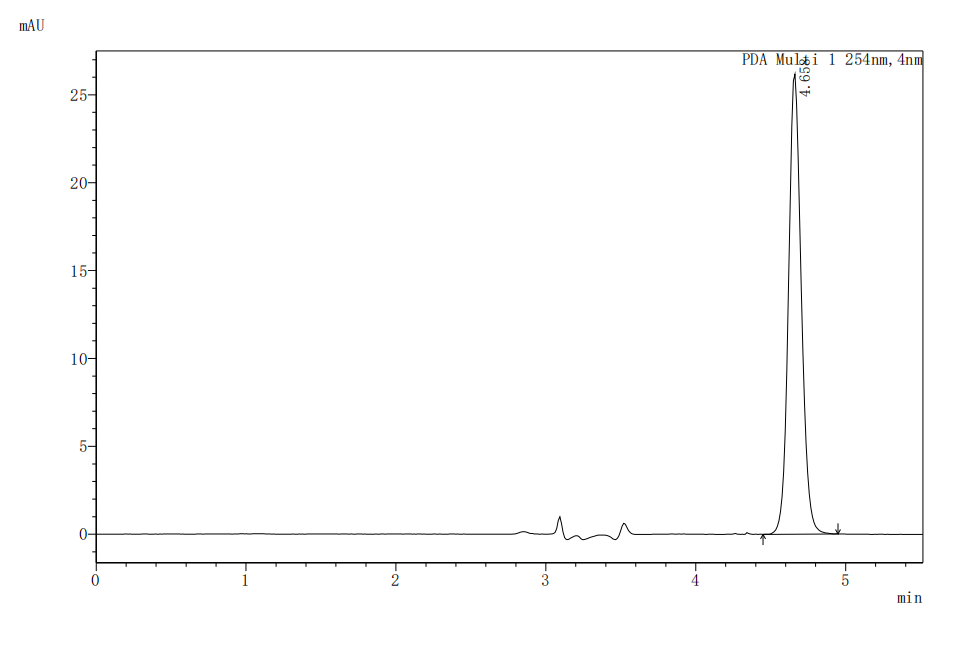A | 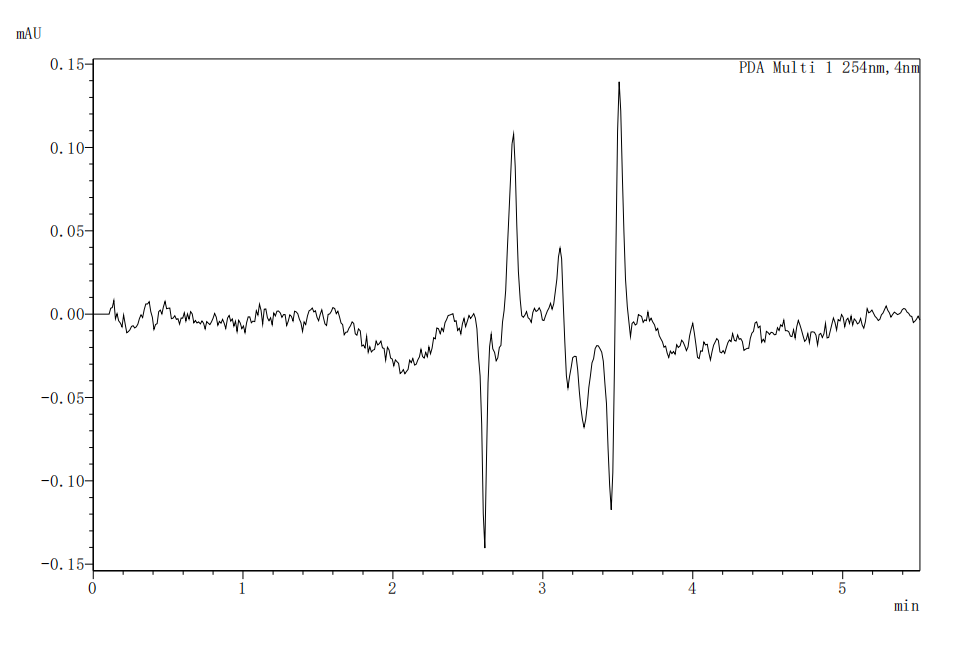B | 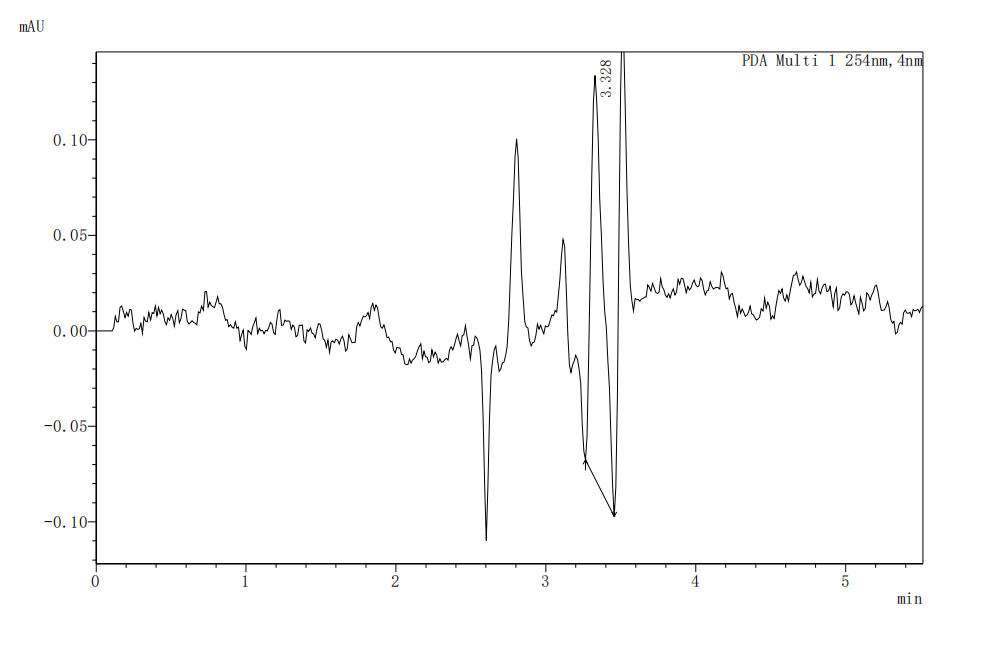C |
| --- | --- | --- |
| 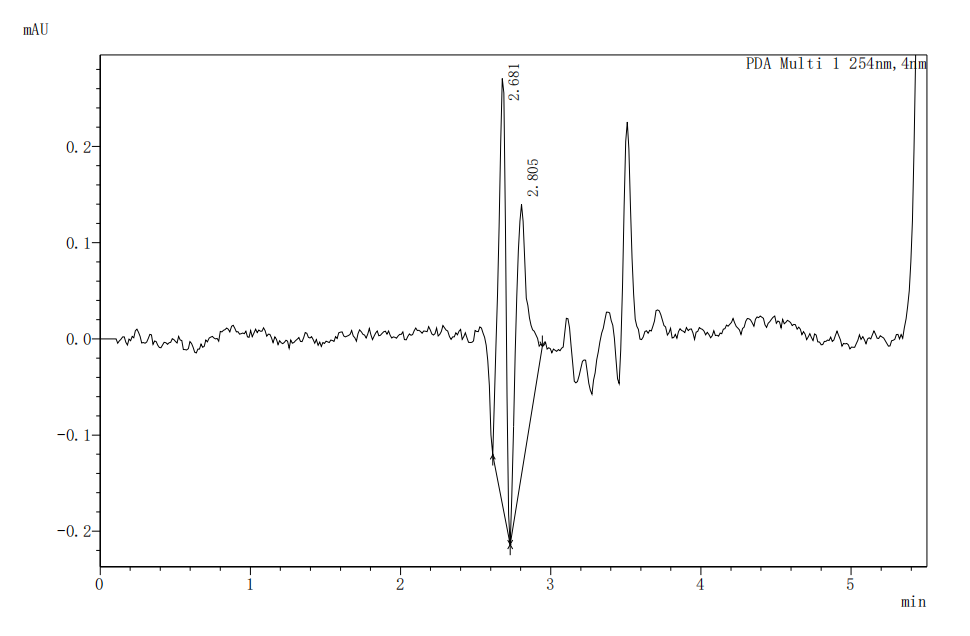D | 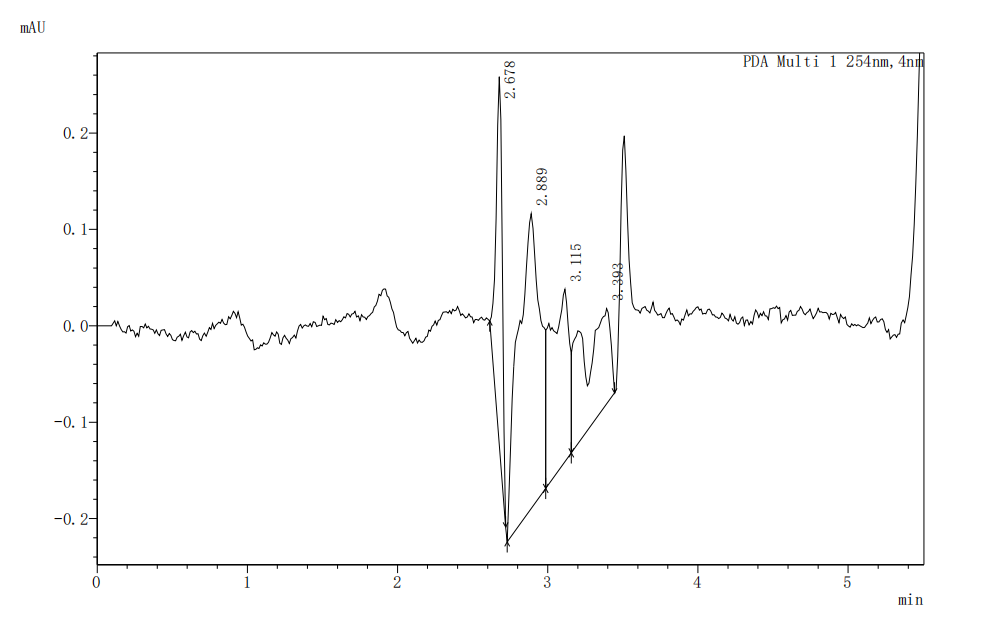E | 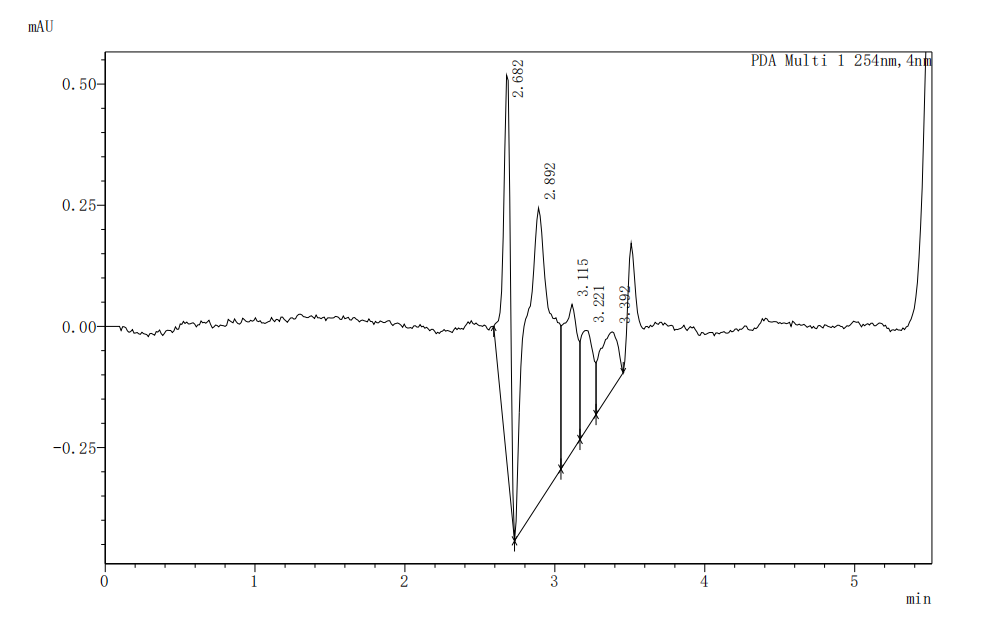F |

Figure S5 A, B, C, D, E, and F respectively represent the chromatograms of metaraminol bitartrate reference standard, 0.9% Sodium Chloride Injection, Sodium Lactate Ringer's Injection, Glucose Sodium Chloride Injection, 5% Glucose Injection and 10% Glucose Injection obtained under the chromatographic conditions corresponding to metaraminol bitartrate.

| 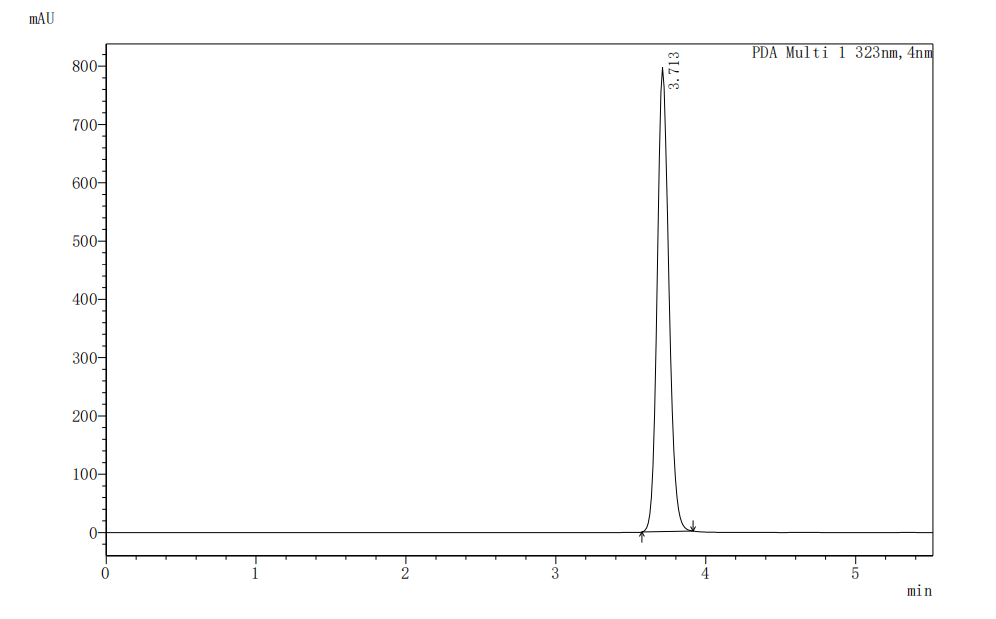A | 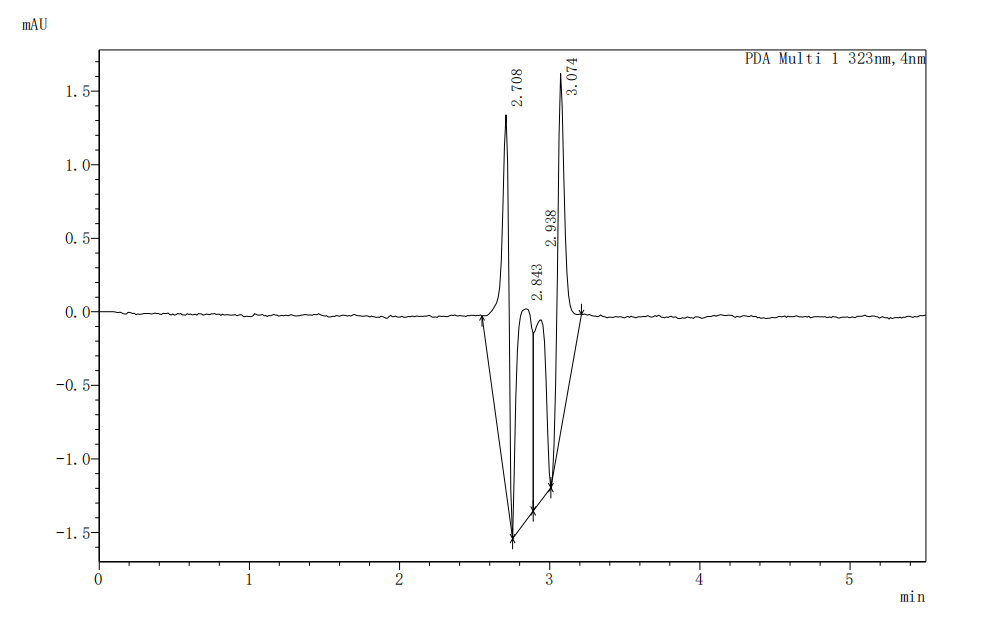B | 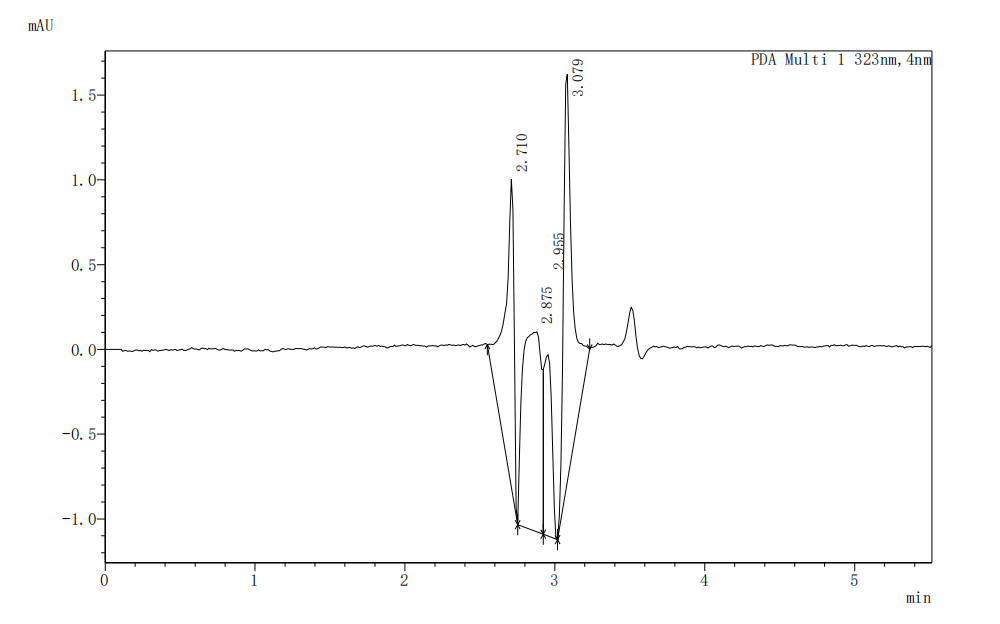C |
| --- | --- | --- |
| 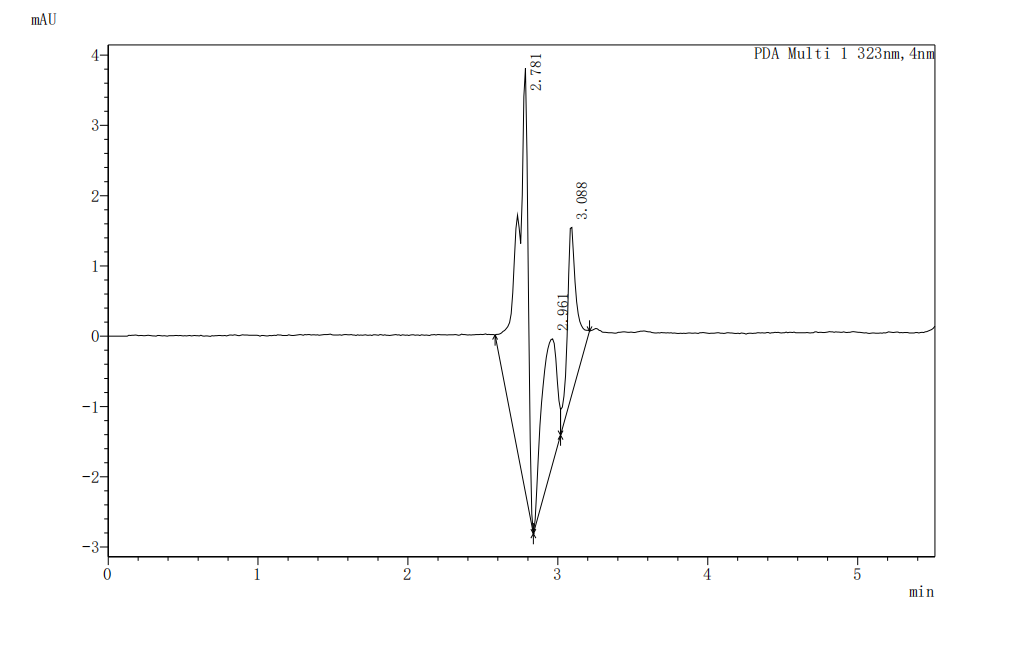D | 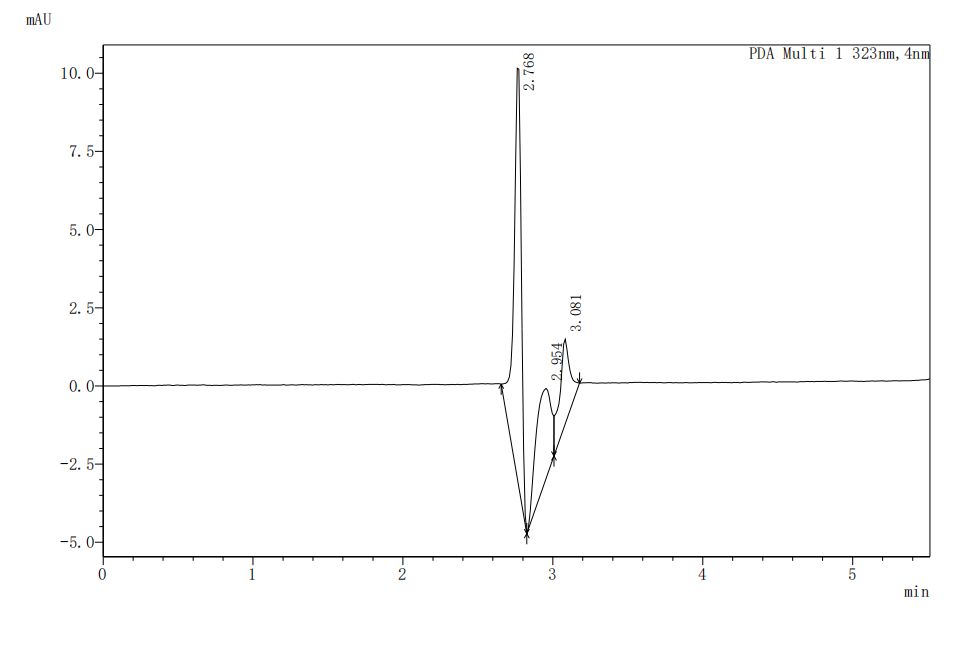E | 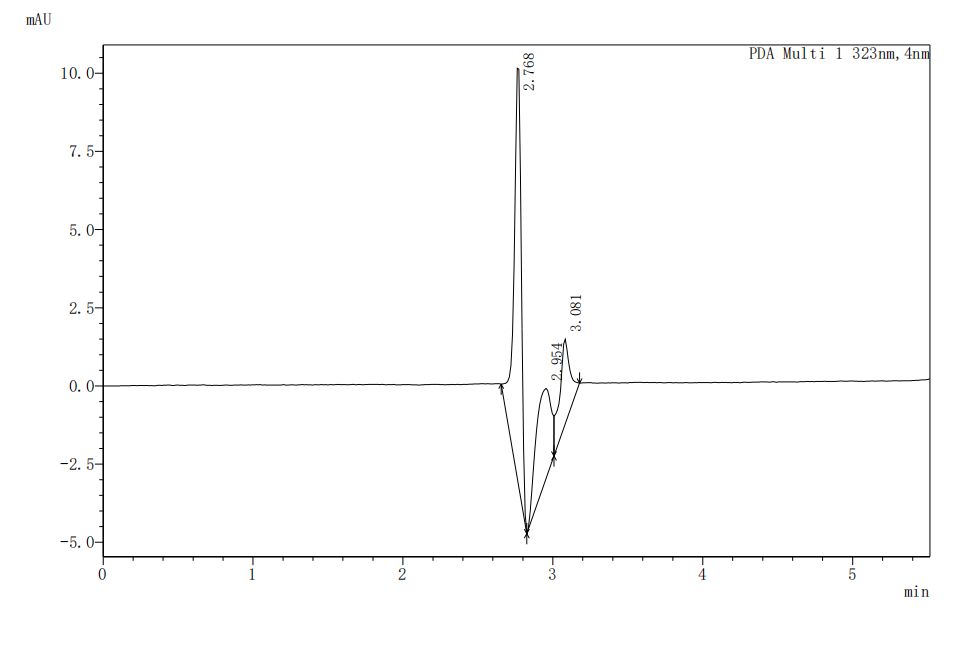F |

Figure S6 A, B, C, D, E, and F respectively represent the chromatograms of milrinone reference standard, 0.9% Sodium Chloride Injection, Sodium Lactate Ringer's Injection, Glucose Sodium Chloride Injection, 5% Glucose Injection and 10% Glucose Injection obtained under the chromatographic conditions corresponding to milrinone.

| 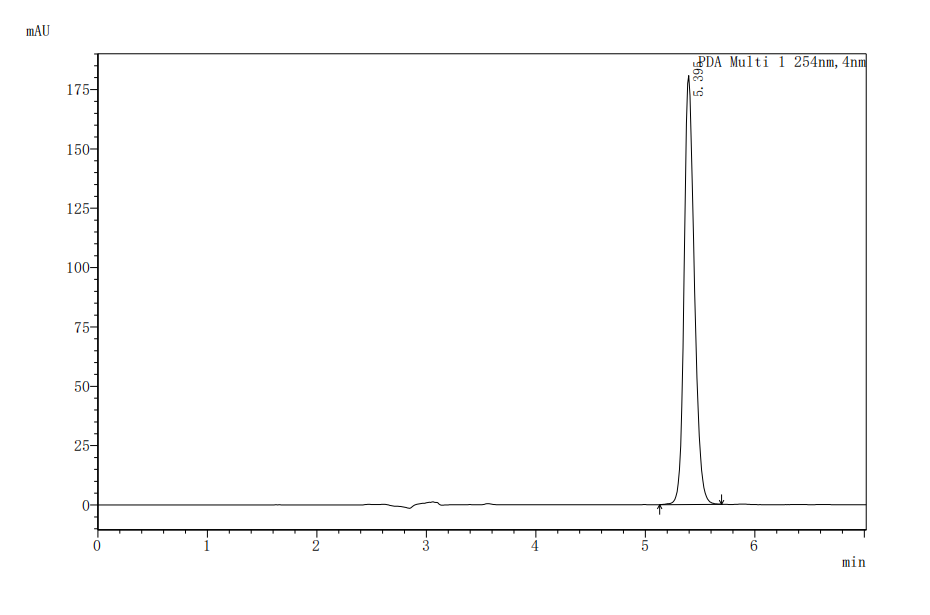A | 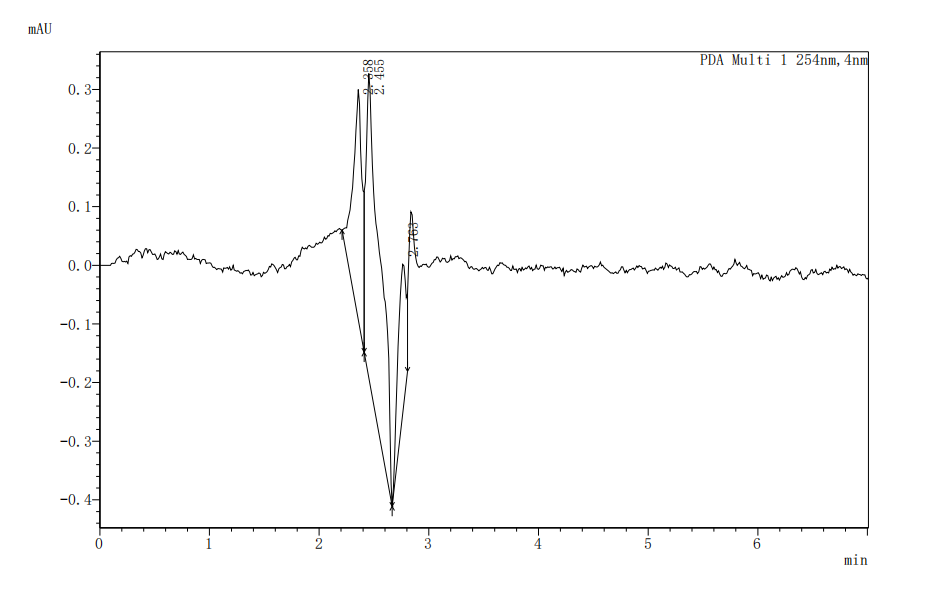B | 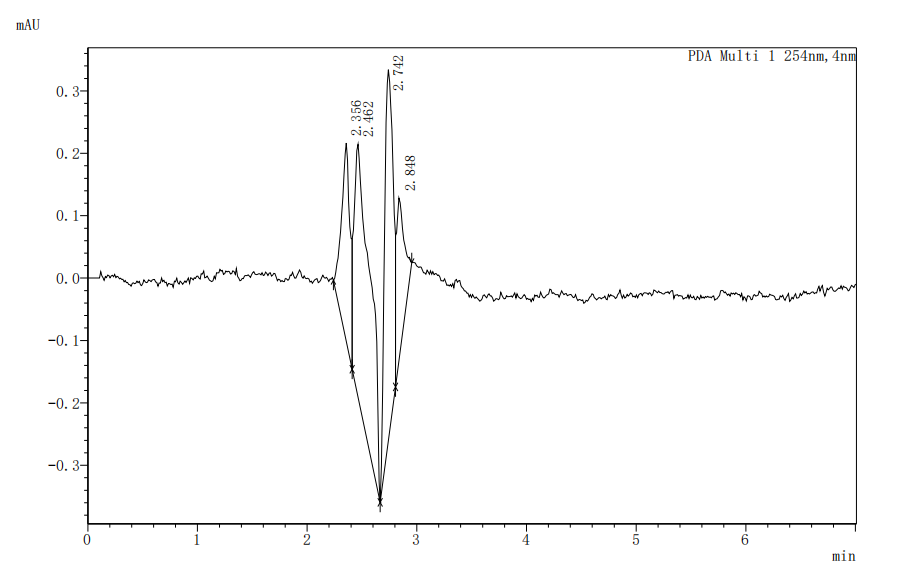C |
| --- | --- | --- |
| 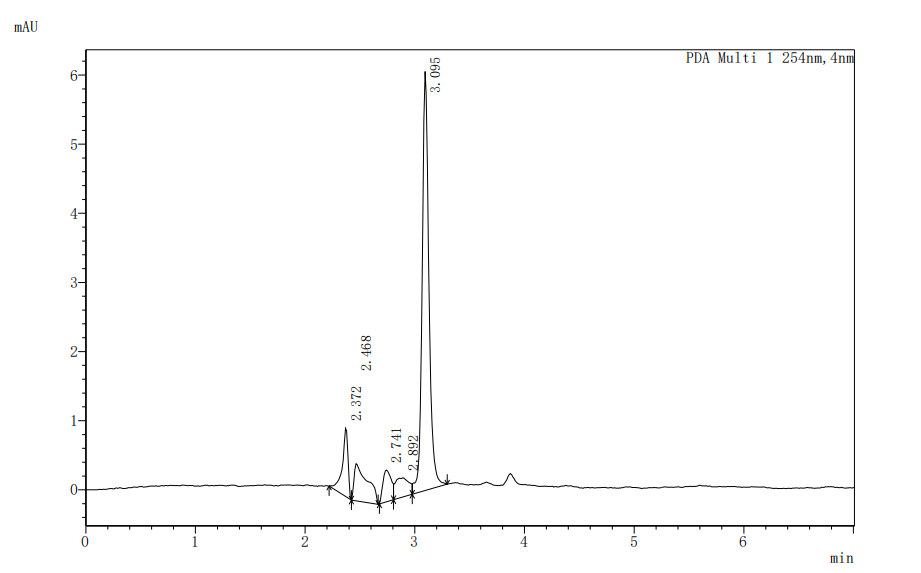D | 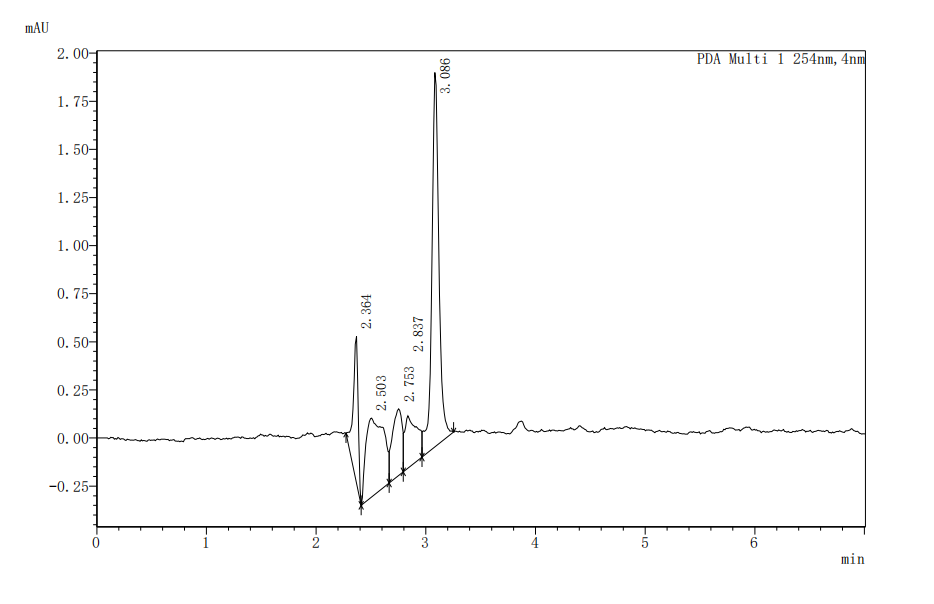E | 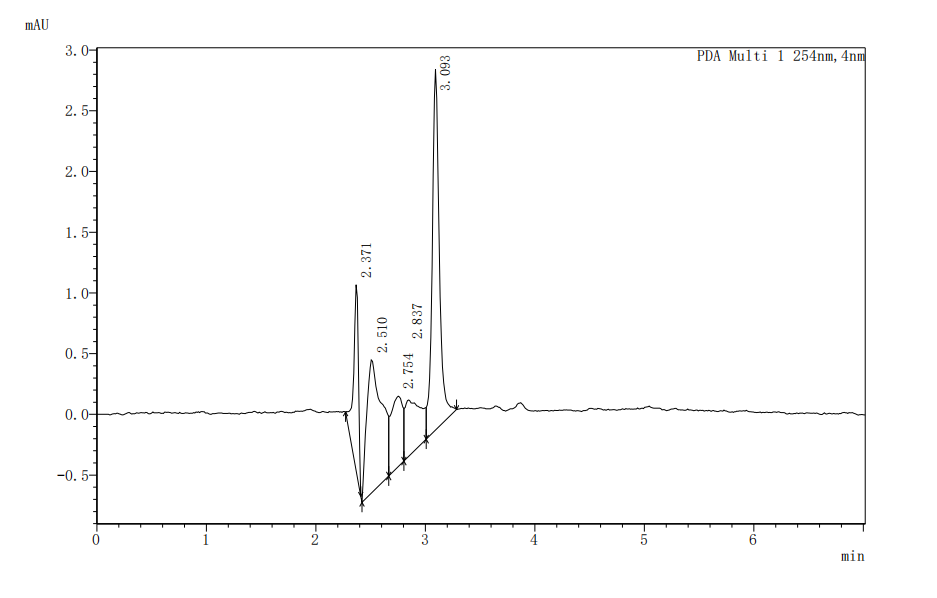F |

Figure S7 A, B, C, D, E, and F respectively represent the chromatograms of nicardipine hydrochloride reference standard, 0.9% Sodium Chloride Injection, Sodium Lactate Ringer's Injection, Glucose Sodium Chloride Injection, 5% Glucose Injection and 10% Glucose Injection obtained under the chromatographic conditions corresponding to nicardipine hydrochloride.

| 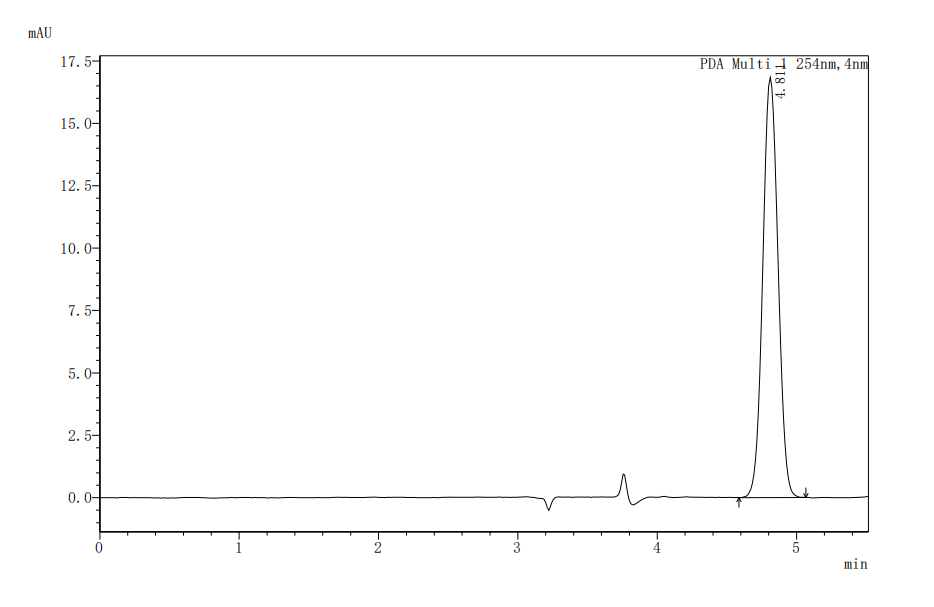A | 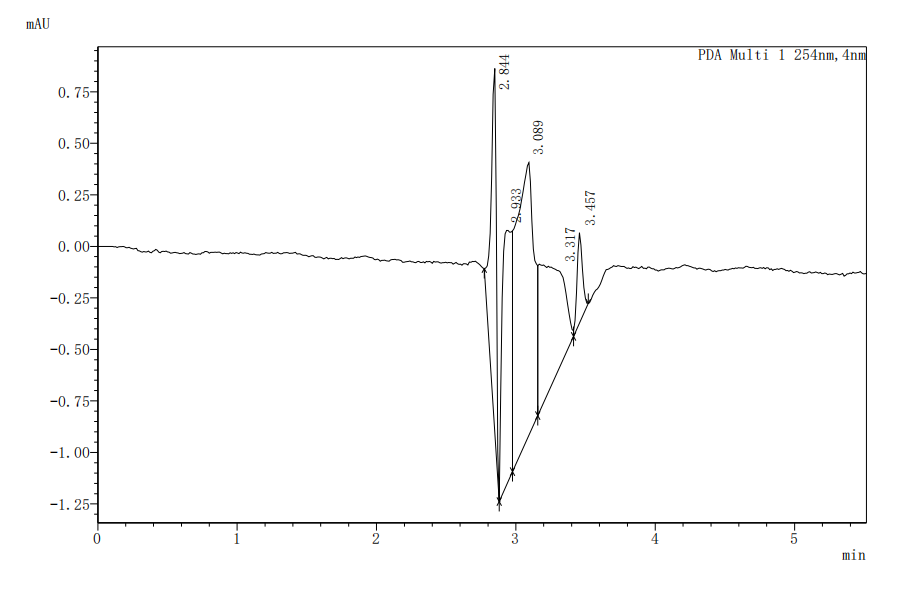B | 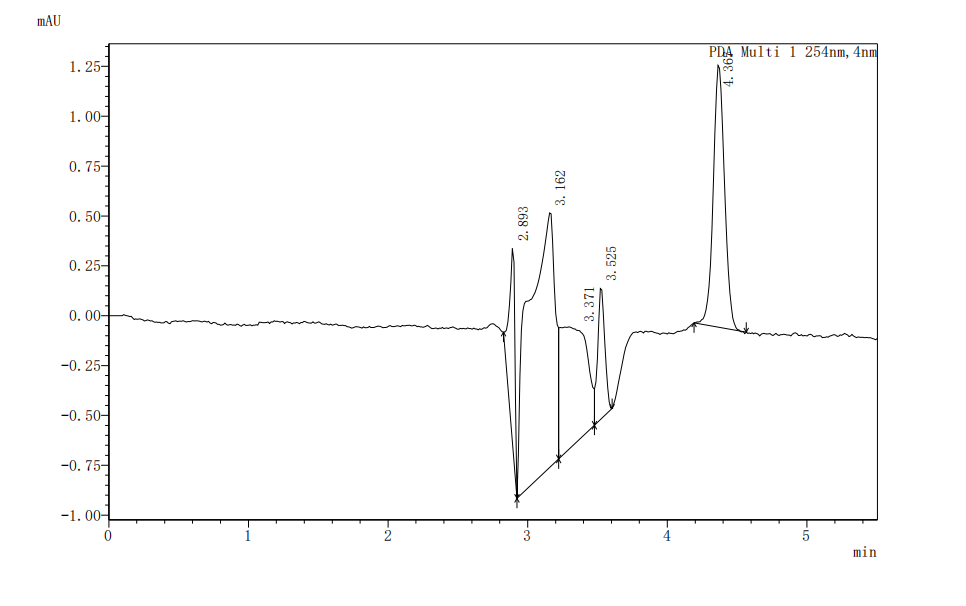C |
| --- | --- | --- |
| 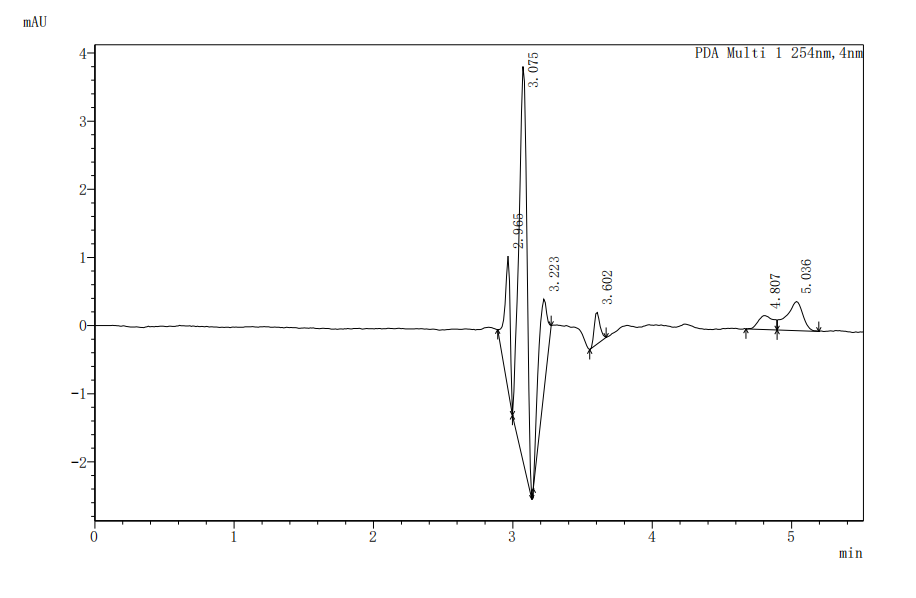D | 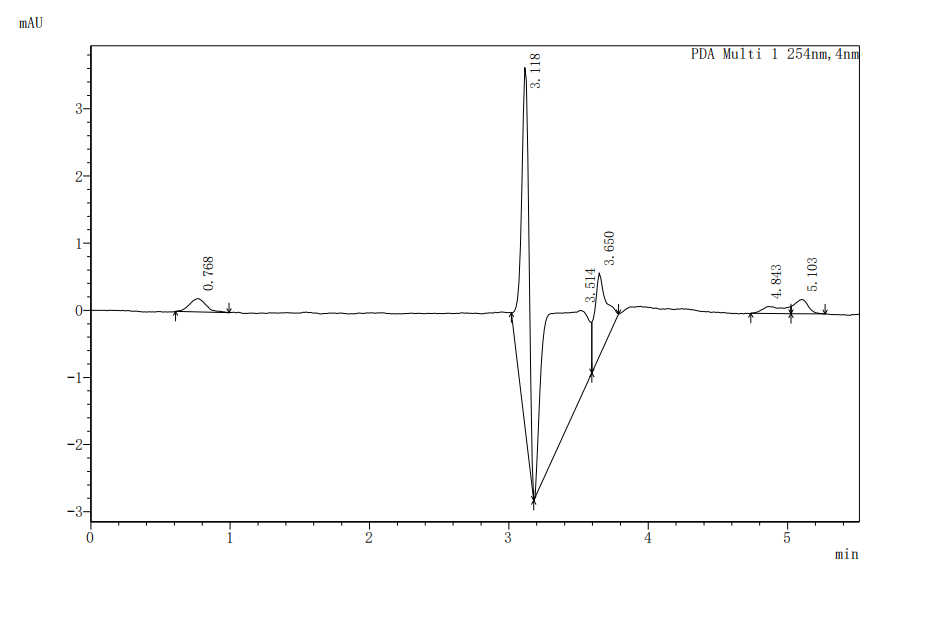E | 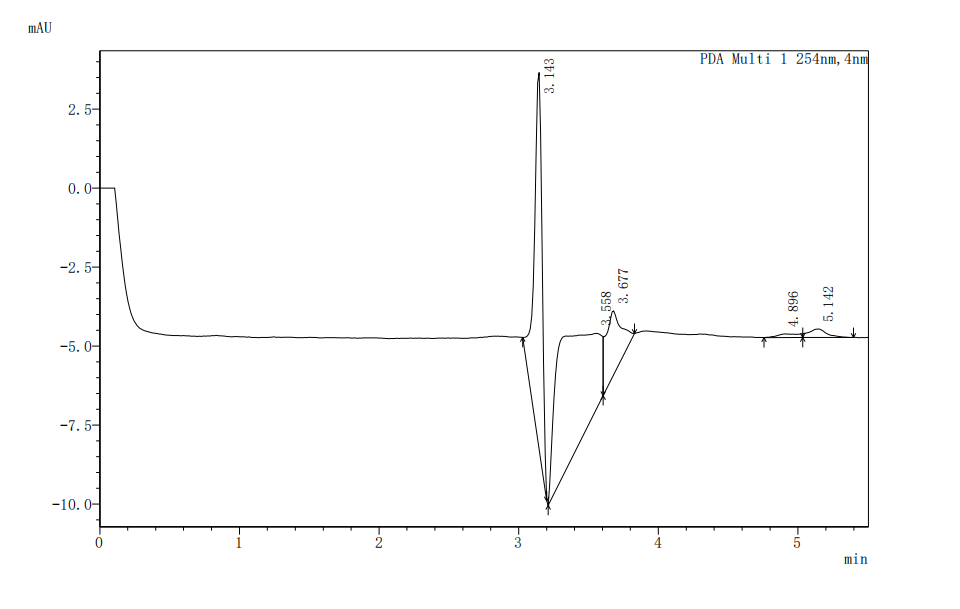F |

Figure S8 A, B, C, D, E, and F respectively represent the chromatograms of epinephrine hydrochloride reference standard, 0.9% Sodium Chloride Injection, Sodium Lactate Ringer's Injection, Glucose Sodium Chloride Injection, 5% Glucose Injection and 10% Glucose Injection obtained under the chromatographic conditions corresponding to epinephrine hydrochloride.

| 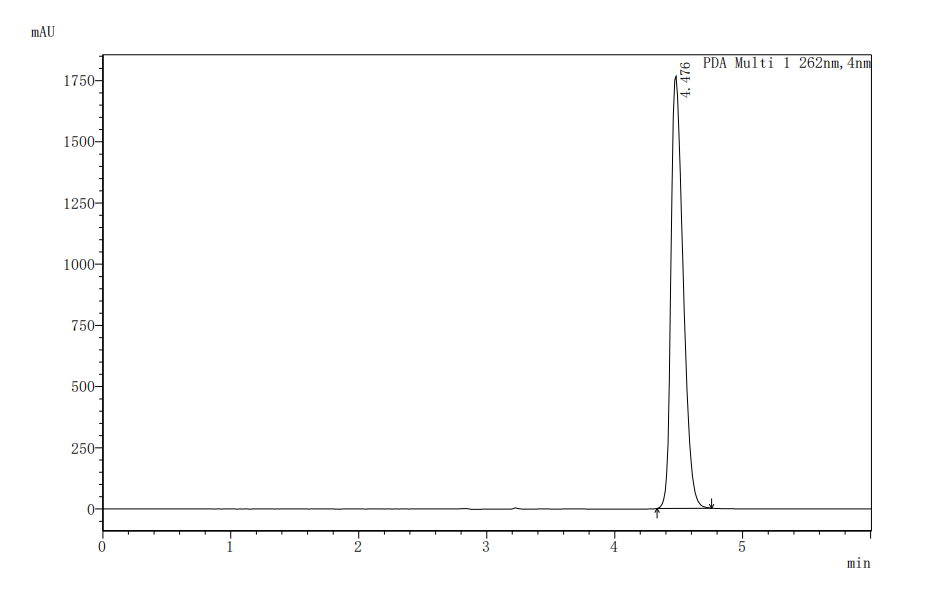A | 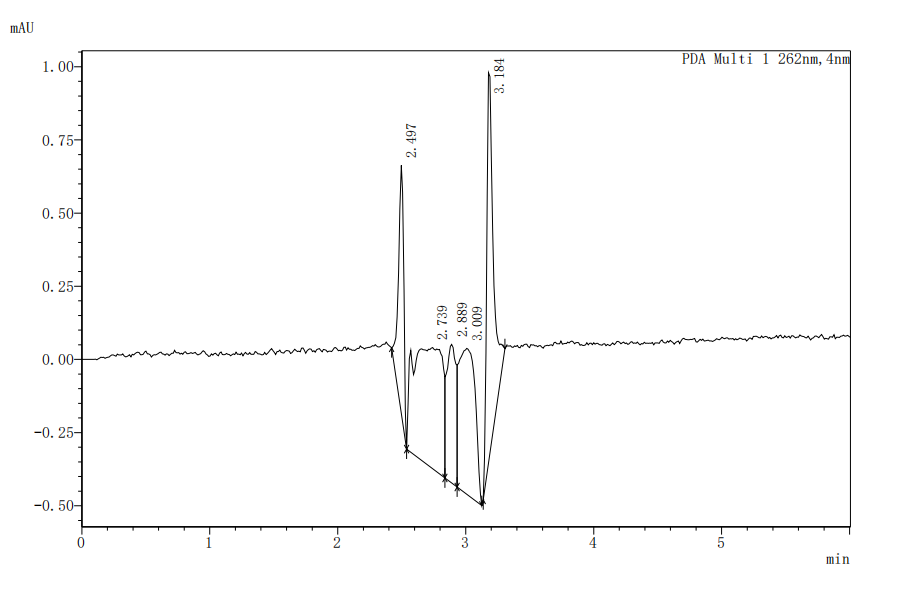B | 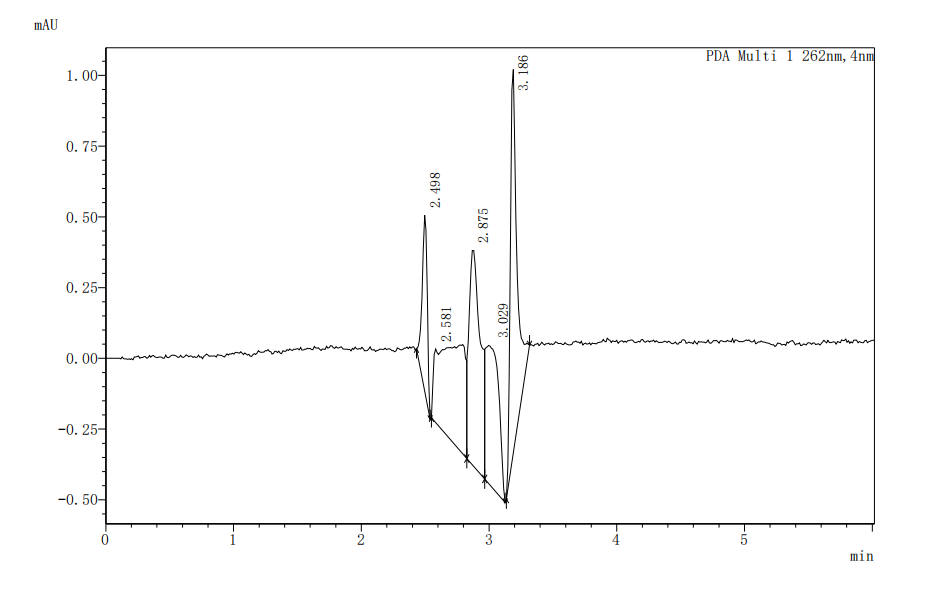C |
| --- | --- | --- |
| 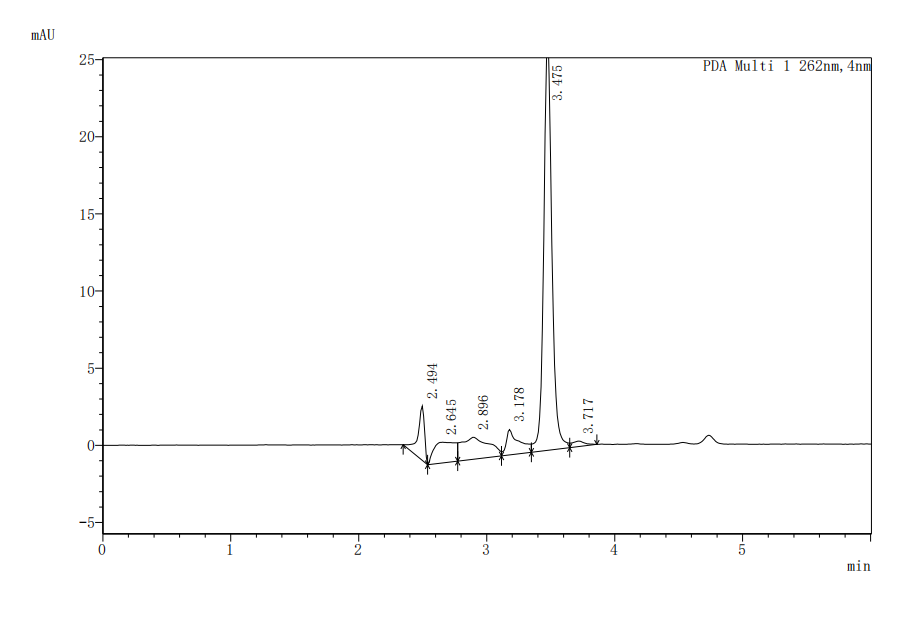D | 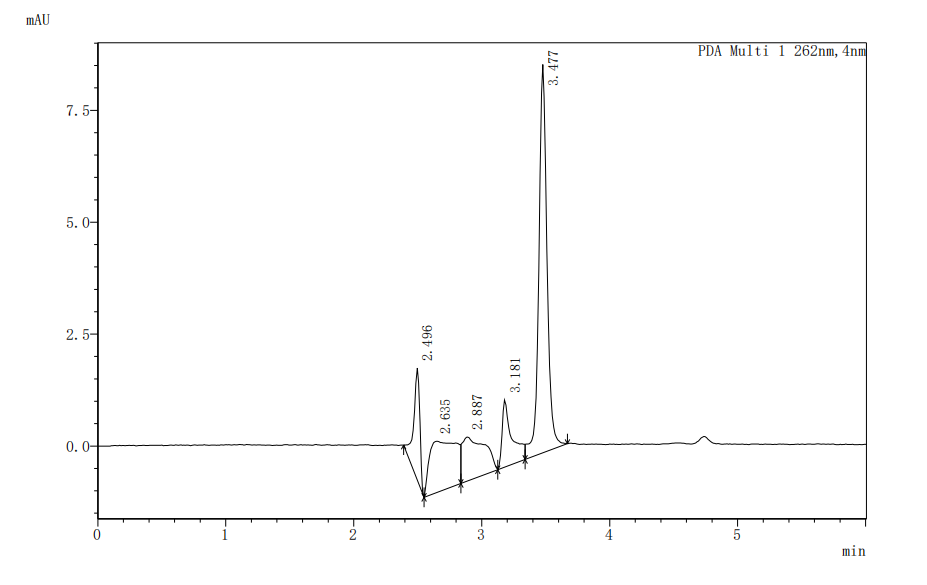E | 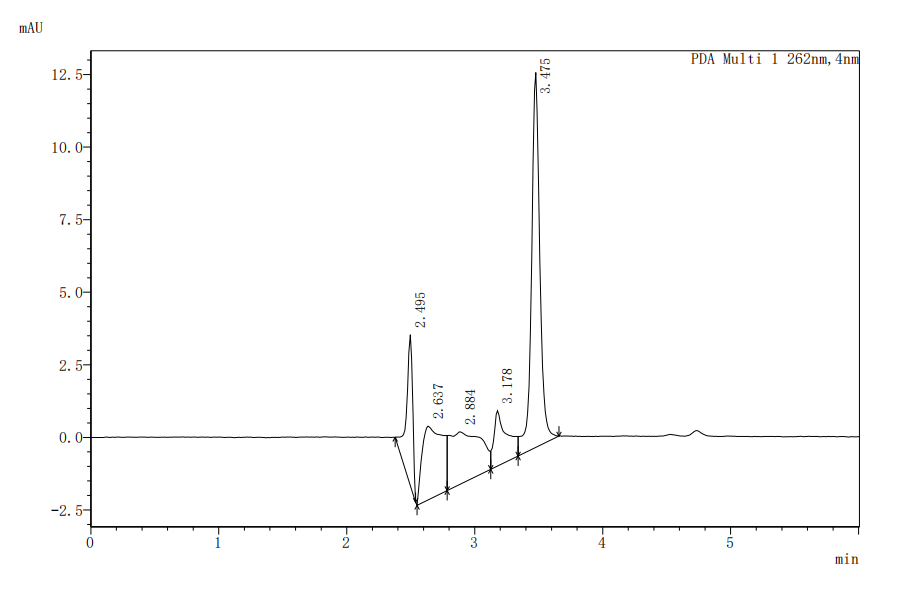F |

Figure S9 A, B, C, D, E, and F respectively represent the chromatograms of urapidil hydrochloride reference standard, 0.9% Sodium Chloride Injection, Sodium Lactate Ringer's Injection, Glucose Sodium Chloride Injection, 5% Glucose Injection and 10% Glucose Injection obtained under the chromatographic conditions corresponding to urapidil hydrochloride.

| 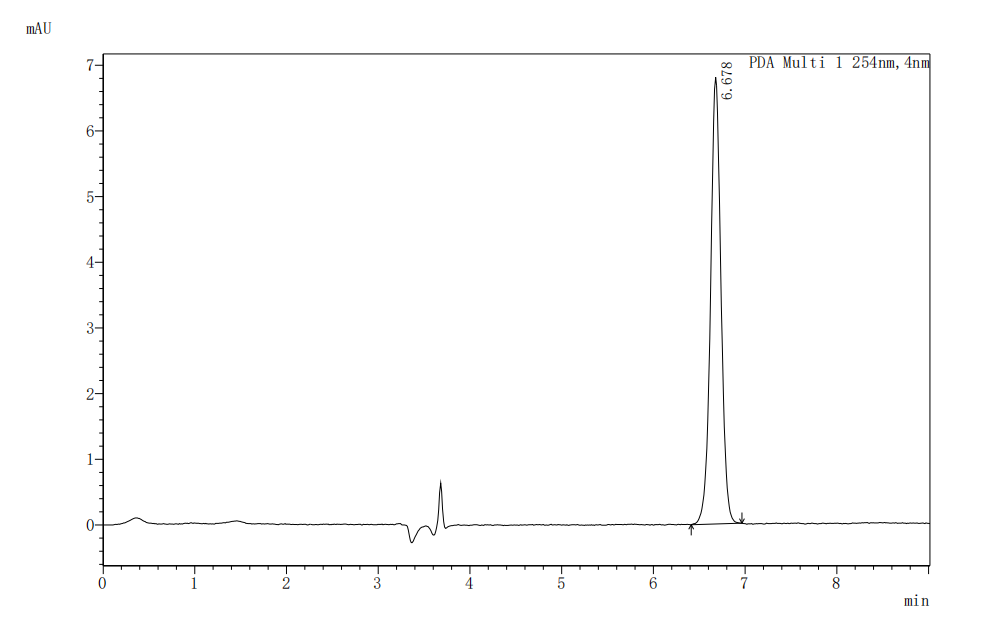A | 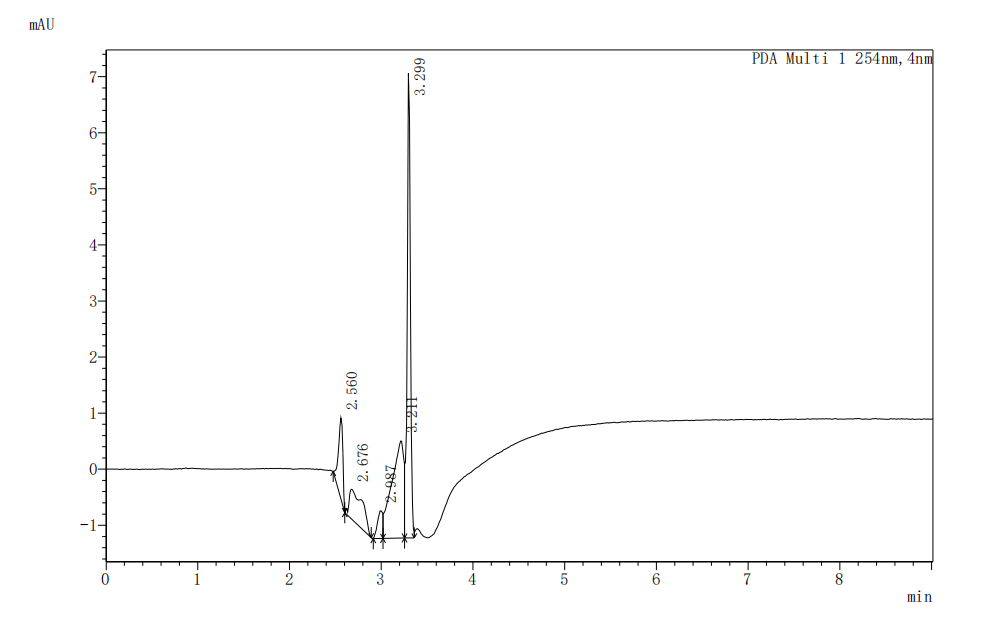B | 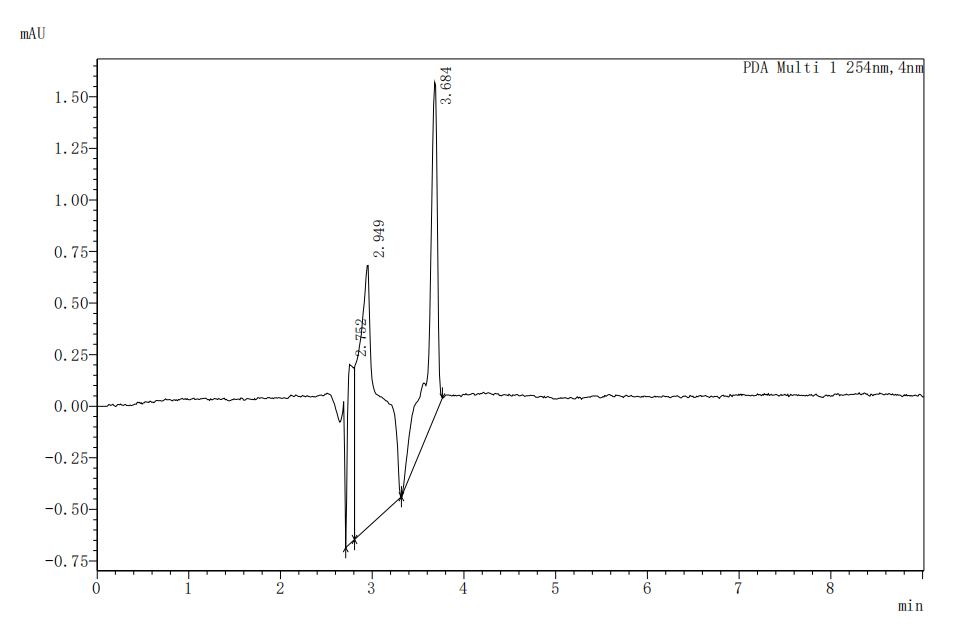C |
| --- | --- | --- |
| 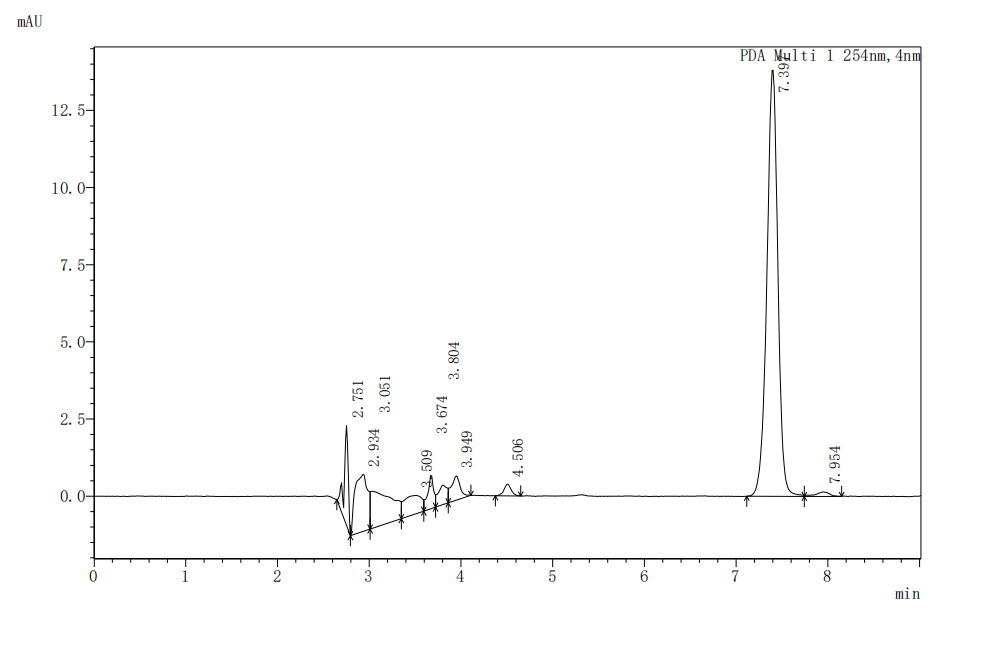D | 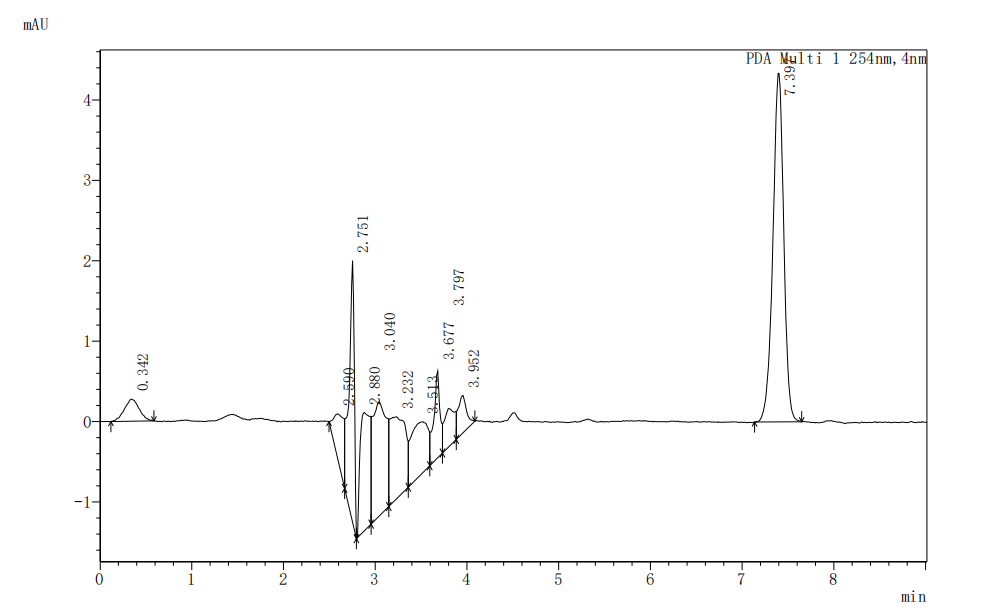E | 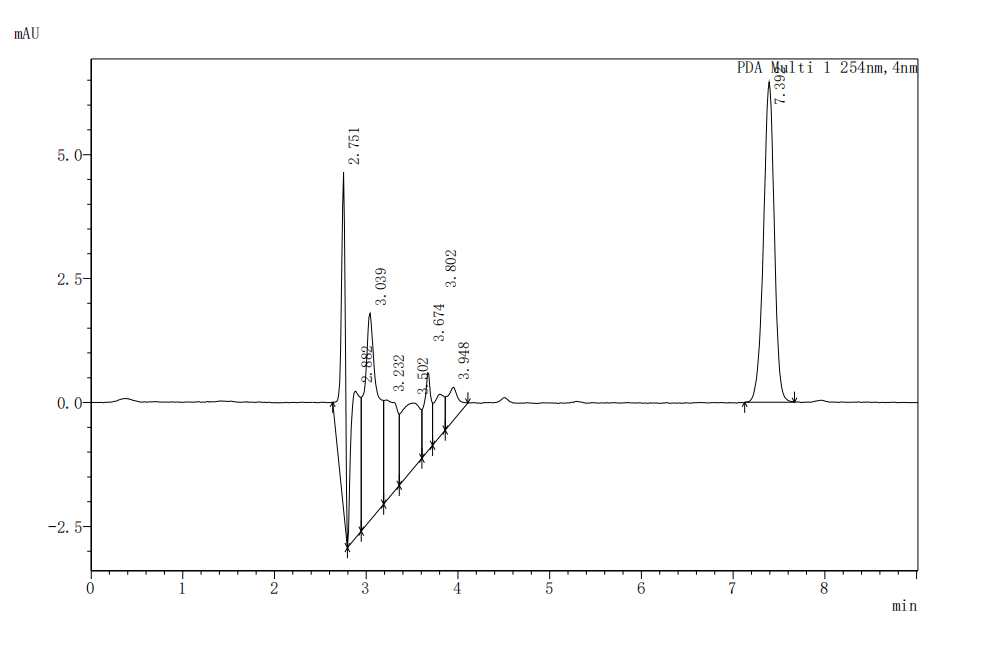F |

Figure S10 A, B, C, D, E, and F respectively represent the chromatograms of isoproterenol hydrochloride reference standard, 0.9% Sodium Chloride Injection, Sodium Lactate Ringer's Injection, Glucose Sodium Chloride Injection, 5% Glucose Injection and 10% Glucose Injection obtained under the chromatographic conditions corresponding to isoproterenol hydrochloride.
